# Iran to achieve the SDG 3.4 at national and sub-national levels

**DOI:** 10.1038/s41598-022-07441-8

**Published:** 2022-03-08

**Authors:** Ali Akbar Haghdoost, Farshad Farzadfar, Moein Yoosefi, Kamyar Mansori, Bagher Larijani, Mohammad Reza Baneshi, Fatemeh Khosravi Shadmani

**Affiliations:** 1 HIV/STI Surveillance Research Center, and WHO Collaborating Center for HIV Surveillance, Institute for Futures Studies in Health, Kerman University of Medical Sciences, Kerman, Iran; 2 Non-Communicable Diseases Research Center, Endocrinology and Metabolism Population Sciences Institute, Tehran University of Medical Sciences, Tehran, Iran; 3 Non-Communicable Diseases Research Center, Endocrinology and Metabolism Population Sciences Institute, Tehran University of Medical Sciences, Tehran, Iran; 4 Department of Biostatistics and Epidemiology, School of Medicine, Zanjan University of Medical Sciences, Zanjan, Iran; 5 Endocrinology and Metabolism Clinical Sciences Institute of Tehran University of Medical Sciences, Tehran, Iran; 6 Modeling in Health Research Center, Institute for Futures Studies in Health, Kerman University of Medical Sciences, Kerman, Iran; 7 Research Center for Environmental Determinants of Health (RCEDH), Health Institute, Kermanshah University of Medical Sciences, Kermanshah, Iran

**Keywords:** Medical research, Risk factors

## Abstract

The present study investigates different scenarios to project the chance of achieving SDG 3.4 in Iran. In this study, the Iranian Death Registry System data was employed to estimate the Unconditional Probability of Dying (UPoD) for the four major categories of NCDs; then, the Bayesian model averaging was used to project the UPoD at the national and sub-national levels. Also, the prevalence of the risk factors was projected by 2030 based on STEPs as well as some other study data. Plus, UPoD and the possibility of achieving the target were estimated once again based on the assumption that the global reduction in risk factors proposed by WHO would be adopted in Iran. The UPoDs for the four NCDs in Iran were 17.5% (95% UI: 16.3–19.2) and 14.7% (13.3–16.2) in 2010 and 2015 respectively and if the current trend continues, 2030 will mark the UPoD of 10.8% (7.9–14.3). However, If the risk factors are reduced to the WHO target level by 2030, the UPoDs will be reduced to 5.44% (3.51–7.39) and 6.55% (5.00–8.13) of the 2010 and 2015 baseline scenarios, respectively, to enable some provinces to meet SDG 3.4. If the current trend continues, Iran will and will not achieve the SDG 3.4 in 2010 and 2015 baseline scenarios, respectively. However, if the global target set for reducing risk factors is achieved, Iran will meet all expectations in SDG 3.4 except in Asthma and COPD. Therefore, effective interventions are recommended to be designed and followed to reduce Asthma and COPD.

## Introduction

All countries in the world are committed to achieve Sustainable Development Goals (SDGs) introduced by the United Nations (UN) in September 2015^1^. The forth target of the third SDGs (SDG 3.4) is focused on Non-Communicable Diseases (NCDs). This target is expected to make a one-third drop in the Unconditional Probability of Dying (UPoD) from NCDs in 30–70 year age group by 2030^1^. World Health Organization (WHO) has, also, developed a global monitoring framework for NCDs along with a set of targets for modifiable risk factors to tackle the global epidemic of NCDs^2^. Bringing these measures into account is necessary to meet SDGs in many countries.

Iran, as a developing country, is experiencing epidemiological transition. These epidemiological changes have led to non-communicable diseases to become the greatest public health problem in the country^3^. This problem will give rise to years of life lost from premature deaths with great impact on life expectancy, and social, political and economic developments^4^. However, we do not possess accurate data on current and future trends of Unconditional Probability of Dying from NCDs at national and provincial levels to trace the progress of achieving SDG 3.4. Also, trends for UPoD may vary by variation of a single risk factor or joint effect of the risk factors. Therefore, to achieve the SDG 3.4, evidence based planning should be strictly carried out. Plus, avoidable number of deaths resulted from reduction of the risk factors, and its impact on UPoD should be modeled and estimated. Given the limited resources, realizing the priorities is imperative for the policy makers to design cost-effective interventions according to the existing resources.

The aim of this study is to project chance of Iran in achieving SDG 3.4 through the different scenarios. These scenarios are based on a reduction of the risk factors to the target levels set by WHO. So far, no study has been conducted on this issue in Iran and only few similar studies have been performed in the world. This paper, additionally, studies the degree to which SDG 3.4 will be achieved at national and sub-national levels by sex. The results can be employed for planning and evidence based policy-making at national and sub-national levels.

## Methods

**Figure 1:**
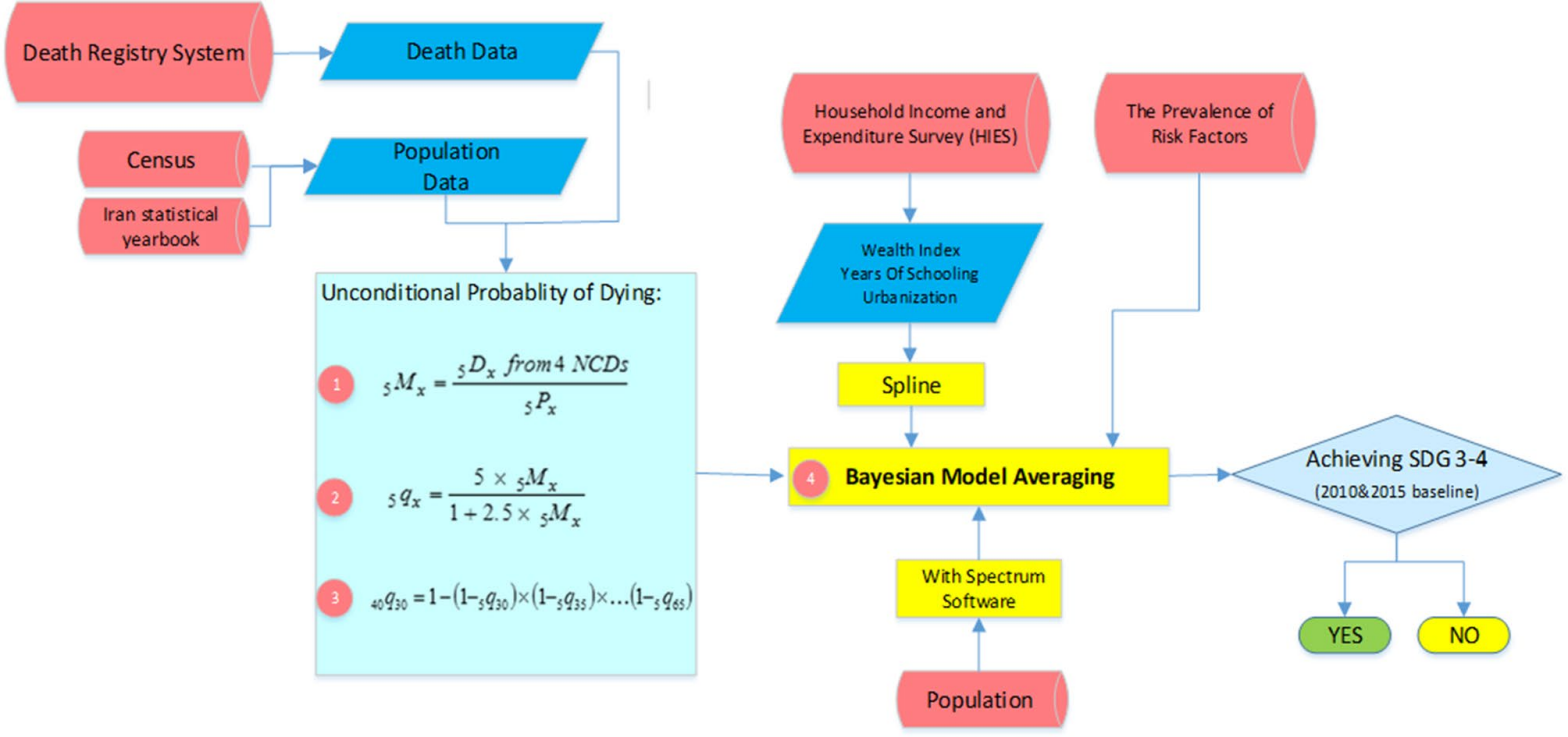
Study workflow in current situation.

**Figure 2:**
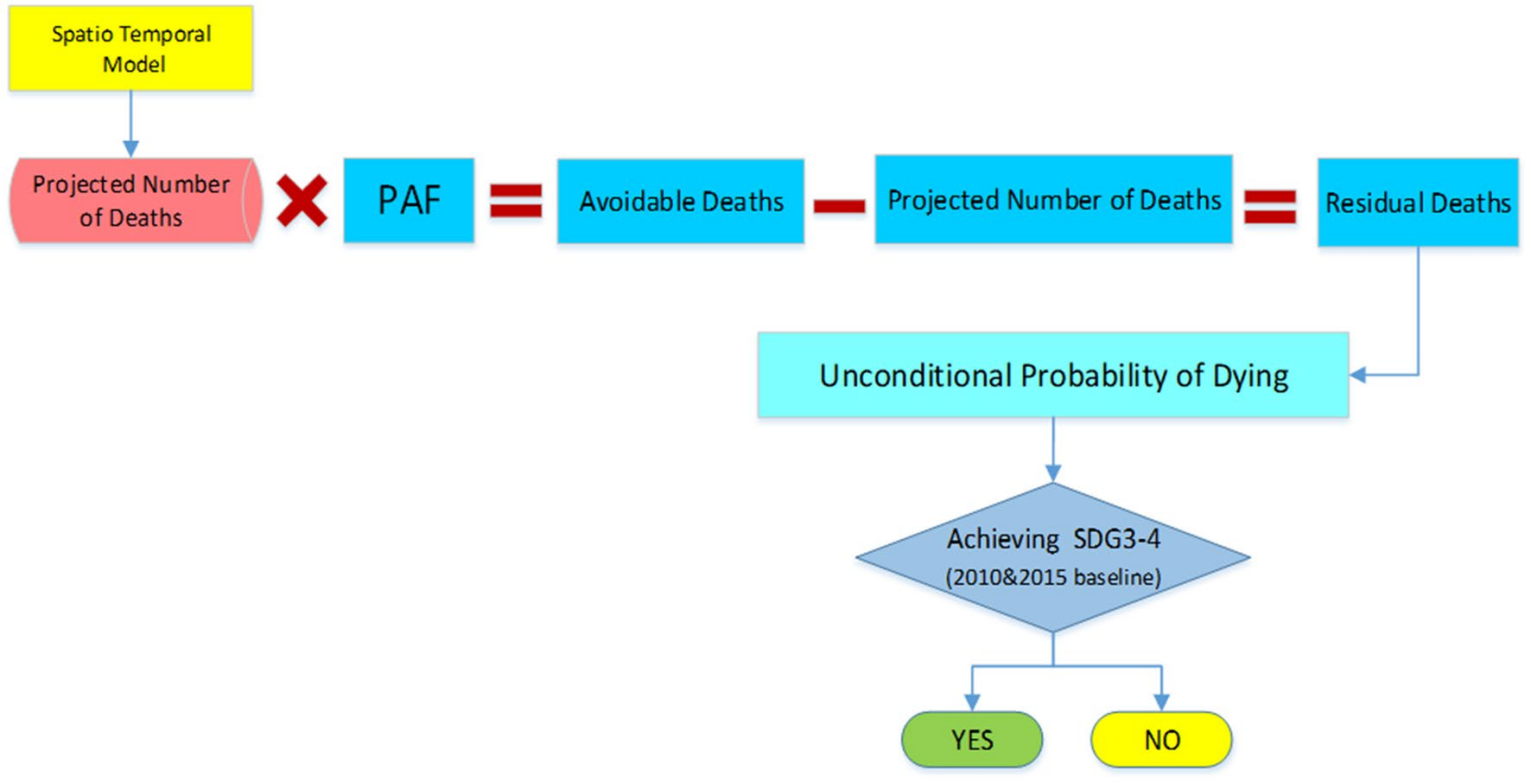
Study workflow in WHO-based scenario.

After addressing the incompleteness and misclassifications in the Iranian Death Registry System (DRS), it was employed to estimate the trends of UPoD for four major categories of NCDs (cancers, cardiovascular diseases, asthma and COPD, and diabetes). Bayesian Model Averaging (BMA) was used to calculate UPoD in the four major NCDs categories and the projection was conducted at the national and sub-national levels by 2030 to estimate the chance of achieving SDG 3.4. In parallel with this, using BMA and Spatio-Temporal models, the prevalence of six major risk factors (Diabetes, Hypertension, Overweight and Obesity, Physical Inactivity, Cigarette Smoking, Salt Intake) were projected by 2030. Avoidable deaths resulted from reducing NCDs risk factors were calculated using Population Attributable Fraction (PAF). UPoD was projected again after reducing avoidable deaths and then the extent of achieving SDG 3.4 at national and sub-national levels was studied in different scenarios. Also, gain in life expectancy after elimination of major NCDs causes of death was estimated by 2030. Study flow is shown in Figs. 1 and 2.

### Definitions

*Unconditional probability of dying (UPOD):* Unconditional probability of dying between ages 30 and 70 years (premature deaths) from NCDs.

*2010 Baseline Scenario*: One third decrease in the UPoD compared to 2010, if current trend continued.

*2015 Baseline Scenario*: One third decrease in the UPoD compared to 2015 if current trend continued.

*2010 WHO based scenario*: One third decrease in the UPoD if the risk factors decreased to the recommended level by WHO compared to 2010.

*2015 WHO based scenarios*: One third decrease in the UPoD if the risk factors decreased to the recommended level by WHO compared to 2015.

### Data sources

#### Death data

We used the Iranian death registration system (DRS). Misclassification and incompleteness of DRS were addressed via Death Distribution Methods. This is discussed in more detail elsewhere^5, 6^. Moreover, redistribution method was adopted to correct the misclassifications in the causes of death and the inconsistency of the causes with age and sex. Also, the records of deaths included many cases of duplicated data that were corrected. Over the course of time, the administrative provincial division in Iran was changed which resulted in an increase in the number of the provinces. Thus, the data was restructured to include the new provinces^7^. To evaluate all-cause mortality rates, the age-spatio-temporal, Gaussian Process Regression (GPR), and Generalized Linear Mixed Models were employed. The method is described in more detail elsewhere^5, 6^.

Due to the modifications done in the registry code and data collection institution before and after 2001, the data before 2001 was not used. The DRS for that period of time (before 2001) did not encompass the cities of Tehran and Isfahan. Therefore, we used information from the cemeteries of Behest-e Zahra in Tehran and Bagh-e Rezvan in Isfahan to complete our datasets. Detailed description has been included elsewhere^7^.

#### Prevalence of the risk factors

The main sources for estimating the trend of prevalence of risk factors was STEPs Surveys carried out for seven rounds in Iran. These surveys were conducted in 2005, 2009, 2011 and 2016. STEPs Surveys included a large sample size to represent Iranian population. Plus, to estimate the prevalence of diabetes, hypertension and obesity, and overweight, some additional studies were employed that had been conducted in Iran (Table 1). The trend of mean of salt intake was obtained from STEPs 2016 and the other Iranian studies (Table1).

**Table 1:** All characteristics of risk factors, WHO targets and theoretical minimum risk exposure level.

Risk factor	Exposure definition	Data source	Related disease	WHO target for risk factors	Theoretical minimum risk exposure level (TMREL)
High systolic and diastolic blood pressure	Mean of systolic and diastolic blood pressure, measured in mmHg	STEPs surveys (from 2005 to 2009–2011–2016) An additional studies: GCS^1^, IHHP^2^, MONICA^3^, NHS^4^,TLGS^5^	Rheumatic heart disease, Hypertensive heart disease, ischemic heart disease, ischemic stroke, hemorrhagic and other non-ischemic stroke, cardiomyopathy, myocarditis, endocarditis, atrial fibrillation and flutter, other circulatory diseases, chronic obstructive pulmonary disease, kidney disease	25% relative reduction in the prevalence of raised blood pressure	110–115 mm Hg
High FPG	Mean of serum fasting plasma glucose measured in mg/dl	STEPs surveys (from 2005 to 2009–2011–2016) An additional studies: GCS, IHHP, MONICA, NHS,TLGS	Diabetes mellitus, Ischemic heart disease, Ischemic stroke, Hemorrhagic and other non-ischemic stroke, Kidney disease	Halt the rise in diabetes	4·8–5·4 mmol/L
Obesity and overweight	Mean of body mass index, measured in kg/m^2^	STEPs surveys (from 2005 to 2009–2011–2016) An additional studies: an additional studies: GCS, IHHP, MONICA, NHS,TLGS	Colon and rectum cancers, liver cancer, pancreas cancer, breast cancer, corpus uteri cancer, ovary cancer, prostate cancer, non-hodgkin lymphoma, other lymphomas and multiple myeloma, leukemia, kidney cancer, gallbladder cancer, thyroid cancer, diabetes mellitus, hypertensive heart disease, ischemic heart disease, ischemic stroke, hemorrhagic and other non-ischemic stroke, kidney disease	Halt the rise in obesity	21–25 kg/m^2^
Physical inactivity	Average weekly physical activity at work, home, transport-related, and recreational measured by MET min per week	STEPs surveys ( from 2005 to 2009–2011–2016)	Colon and rectum cancers, breast cancer, diabetes mellitus, ischemic heart disease, ischemic stroke	10% reduction in the prevalence of physical inactivity	All adults experience 3000–4500 MET min per week
Smoking	Daily smoking Smoking Impact ratio(SIR)	STEPs surveys (from 2005 to 2009–2011–2016)	Mouth and oropharynx cancers, esophagus cancer, stomach cancer, colon and rectum cancers, liver cancer, pancreas cancer Trachea, bronchus and lung cancers, melanoma and other skin cancers, breast cancer, cervix uteri cancer, corpus uteri cancer, ovary cancer, prostate cancer, bladder cancer, non-hodgkin lymphoma, multiple myeloma, other lymphomas and multiple myeloma, leukemia, kidney cancer, gallbladder cancer, thyroid cancer, diabetes mellitus, rheumatic heart disease, hypertensive heart disease, ischemic heart disease, ischemic stroke, hemorrhagic and other non-ischemic stroke, chronic obstructive, pulmonary disease, asthma, other respiratory diseases	30% relative reduction in the prevalence of current tobacco use	All individuals are lifelong non-smokers
Salt intake	24 h urinary sodium measured in gram per day Mean of salt intake	STEPs in 2016 Comprehensive project on household food pattern and nutritional status, TLGS, IHHP, Isfahan salt study, PGHHS^6^, Mashhad Study, YHHP^7^, Urban Health Equity assessment and Response Tool (Urban Heart-phase1), HIEs^8^	Direct : stomach cancer Indirect : rheumatic heart disease, hypertensive heart disease, ischemic heart disease, ischemic stroke, hemorrhagic and other non-ischemic stroke, cardiomyopathy, myocarditis, endocarditis, atrial fibrillation and flutter, other circulatory diseases, chronic obstructive pulmonary disease, kidney disease	30% reduction in the mean population intake of salt	24 h urinary sodium between 1 and 5 g per day

1. Golestan Cohort Study.

2. Isfahan Healthy Heart Program.

3. Monitoring trends and determinants in cardiovascular disease.

4. National Health Survey.

5. The Tehran Lipid and Glucose Study.

6. Persian Gulf Healthy Heart Study.

7. Yazd Healthy Heart Program.

8. Household Income and Expenditure survey.

#### Relative risk (RR)

RRs were extracted from the Global Burden of Disease (GBD) and NCD.RisC (NCD risk factor collaboration)^8–11^. These RRs were derived for each risk factor-disease pair and then were categorized by age and sex.

#### Population data

The population data came from censuses carried out in 1996, 2006, 2011, and 2016, as well as the Iranian statistical yearbook divided by sex, age and province. Spectrum software (version 5.72) was employed to project population by 2030.

#### Covariate data

We used household expenditure and income survey to estimate the variables of the wealth index, years of schooling, and urbanization until 2015 sorted by age, sex, and province of residence^12^. Also, the spline method was used to estimate these covariates of wealth index, years of schooling, and urbanization until 2030. Then the results were entered into the covariate model.

### Statistical analysis

#### Unconditional probability of dying

UPoD was calculated based on WHO guidelines from 2001 to 2015^13^. Also, Bayesian Model Averaging (BMA) was used for its projection. According to this model, the ten covariates (six risk factors, wealth index, years of schooling, the percent of urbanization and population) produced 1024 different models (2^10^ = 1024). BMAs in all of the models were integrated and with regard to the marginal likelihood of each model, they were weighted. In this model, prior distribution was assumed to be uniform. The number of burn-ins, and iterations were respectively retained to be 5000 and 20,000. Plus, selecting the baseline for comparison was optional. In this study, we considered 2010 and 2015 the two baselines to conduct comparisons and project SDG 3.4 achievement status (2010 and 2015 baseline scenarios).

#### Risk factors

The prevalence of diabetes, hypertension and obesity, and overweight was estimated with Gaussian Process Regression (GPR). more details are available elsewhere^14, 15^.The prevalence of physical inactivity and smoking was extrapolated from 2001 to 2005 and the missing points were interpolated in with spatiotemporal model. The data for two risk factors are also available in 2000. We do not possess any data for the year of 2001 to 2005. The trend of prevalence of risk factors was projected with BMA and spatiotemporal models (more details in supplementary appendix 1).

#### Population attributable fraction (PAF)

PAF was calculated for each pair of the risk factor-disease (supplementary appendix 2) in different scenarios (2010 and 2015 baselines) by sex and province. Risk factor scenarios are presented in Table 1. Joint effect of risk factors was calculated as well (Eq. 1). Mortality rate by 2030 was projected with spatio-temporal model by sex, cause and province.

1 $$PAF = 1 - \prod\limits_{i = 1}^{n} {\left( {1 - PAF_{i} } \right)}$$

Then the number of deaths from the multiplication of the death rate in the population was calculated. After subtracting the avoidable deaths from projected deaths, the UPoD was calculated again.

#### Cause deleted life table

The impact of reducing major NCDs related causes of deaths on increasing life expectancy was estimated by cause-deleted life table and the composition method.

## Results

### Current situation

According to the results of our study, if the existing trend continues based on the 2010 baseline, a one-third reduction in UPoD will be achieved by 2030 (however, if the trend is broken down to the categories of NCDs, the trends of CVDs and diabetes, as two separate trends, will be very close to reaching the target but won’t meet the expected levels). On the flip side, there will be no reduction of risk factors in 2015 baseline scenario. The values of UPoD from cancers in Iran were 4.2% (95% UI: 3.8–4.6) and 3.2% (2.9–3.6) in 2010 and 2015 respectively. This value is projected to be 2.2% (1.5–3.1) by 2030.

**Figure 3:**
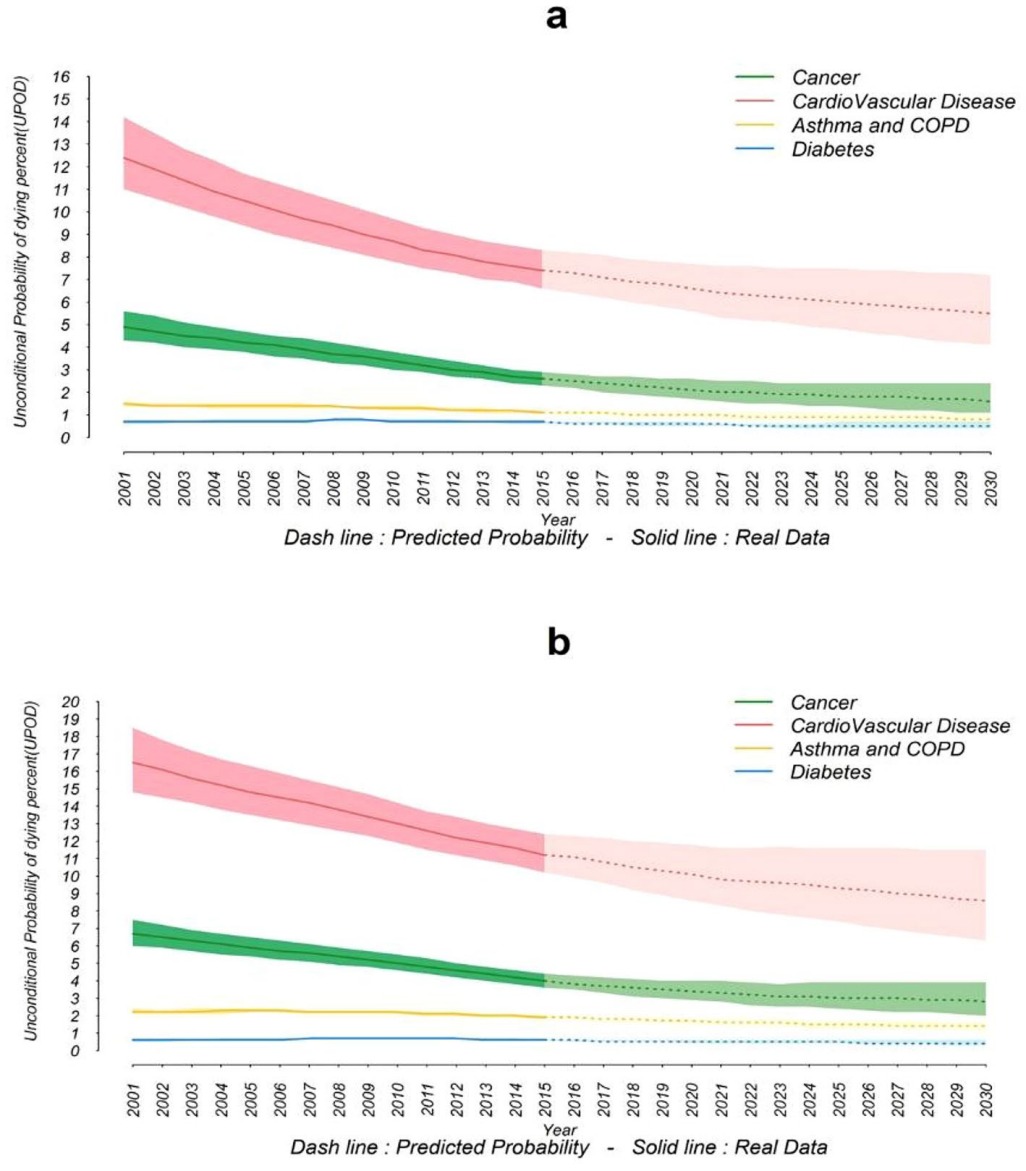
Trends and projections of unconditional probability of dying in four categories of NCDs. (**a**) Females, (**b**) Males.

The corresponding values for 2010 baseline scenario, 2015 baseline scenario and the overall 2030 projected value for CVDs are 10.8% (9.8–12.0), 9.3% (8.3–10.3.), and 7.0% (5.1–9.3) respectively. The UPoDs from asthma and COPD in females were 1.7% (1.6–1.8) and 1.5% (1.4–1.6) in 2010 and 2015 respectively; and the overall asthma and COPD related UPoD is projected to drop to 1.0% (0.8–1.3) by 2030. The corresponding values for diabetes are 0.7% (0.6–0.7), 0.6% (0.5–0.6), and 0.4% (0.3–0.6) respectively. The trends and projections of UPoD in the 2001 to 2030 time-frame, are shown in Fig. 3a by categories. The trends and projections of UPoD for the years of 2001 to 2030 are indicated in Fig. 3 by sex and categories of NCDs.

### Prevalence of risk factors

Based on our projections, the age-standardized prevalence of diabetes, hypertension, obesity and overweight, and physical inactivity trend from 2015 to 2030 will increase in females by17%, 41, 32% and 29% respectively. The corresponding values in males will be 20%, 41%, 48% and 39%. The prevalence of smoking will be decreased by 27% in females and 28% in males in the same period. Also, mean of salt intake will be dropped by 12% and 16% in females and males, respectively.

The age-standardized prevalence and projection of diabetes, hypertension, obesity and overweight, physical inactivity, smoking, and mean of salt intake are shown in Table 2 in three time periods. The prevalence of risk factors at sub-national level are provided in supplementary appendix 3.

**Table 2:** Prevalence of risk factors and mean of salt intake in three time periods by sex.

Risk factor	2010		2015		Projected by 2030	
	F	M	F	M	F	M
Diabetes (%)	8.9 (8.4–9.3)	7.3 (6.9–7.8)	9.5 (9–9.9)	7.9 (7.5–8.4)	11.2 (10.8–11.7)	9.5 (9.0–10.0)
Hypertension (%)	25.4 (23.2–27.5)	21.0 (19.3–22.7)	30.0 (27.9–32.1)	24.8 (23.1–26.4)	42.5 (39.0–46.1)	35.2 (32.1–38.5)
Obesity (%)	27.3 (25.0–29.6)	14.3 (12.9–15.8)	32.2 (30.1–34.1)	17.7 (16.3–19.1)	42.6 (40.2–45.2)	26.3 (23.8–28.9)
Physical inactivity (%)	50.4 (46.4–54.2)	38.5 (34.5–42.3)	58.6 (55.1–61.9)	46.8 (43.1–50.2)	75.9 (71.9–79.9)	65.5 (61.1–70.2)
Smoking (%)	3.7 (3.7–3.7)	27.1 (25.2–29.0)	3.3 (3.3–3.3)	24.3 (22.4–26.3)	2.4 (2.4–2.4)	17.6 (15.6–19.5)
Mean of salt intake (grams per day)	9.6 = − (9.5–9.8)	10.4 (10.2–10.6)	9.3 (9.1–9.5)	9.9 (9.7–10.1)	8.2 (7.6–8.8)	8.3 (7.7–8.9)

### Avoidable deaths by 2030

According to the projected mortality rate and the prevalence of risk factors in 2030, avoidable deaths from the risk factors are categorized and estimated in three different scenarios. The number of avoidable deaths by sex, cause, and risk factor are shown in Table 3. The number of avoidable deaths at sub-national level is provided in supplementary appendix 4. The projected number of deaths in 2030 will be 47,847 among females and 64,784 among males in the 30–70 age group. The number of avoidable deaths from joint effects of risk factors in theoretical minimum exposure risk level (TMREL) will be 42,928 in females and 55,578 in males. The corresponding numbers in WHO-based scenarios will be 30,638 and 37,598 in females and males in 2010 baseline scenario. These same values in 2015 baseline scenario are calculated to be 24,853 and 29,566 in WHO-based scenario in females and males respectively.

**Table 3:** Avoidable deaths by NCDs categories with reducing the risk factors by 2030.

Risk factor	Theoretical minim exposure risk level scenario^a^								WHO-based scenario10^b^								WHO-based scenario15^c^							
	Cancers		CVDs		Asthma and COPD		Diabetes		Cancers		CVDs		Asthma and COPD		Diabetes		Cancers		CVDs		Asthma and COPD		Diabetes	
	F	M	F	M	F	M	F	M	F	M	F	M	F	M	F	M	F	M	F	M	F	M	F	M
Diabetes	–		10,443	18,611	–	–	2455	2286	–	–	473	1159	–	–	1044	884	–	–	145	467	–	–	702	608
Hypertension	–	– scenario 1	27,798	26,026	–	–	95	91	–	–	9342	9746	–	–	35	24	–	–	8968	9186	–	–	33	23
Obesity	1949	1004	7622	9550	–	–	1964	1526	752	438	2085	3185	–	–	451	495	277	217	1649	2888	–	–	168	212
Physical inactivity	1089	832	1003	326	–	–	4545	7151	798	365	3591	7943	–	–	866	931	606	276	2737	6027	–	–	658	710
Smoking	1649	757	7435	4482	678	385	180	53	158	322	918	1521	77	135	12	40	173	349	1010	1648	84	147	14	44
Mean of salt intake	334	594	10,947	17,961	–	–	–	–	83	151	6267	8566	–	–	–	–	117	219	4612	5247	–	–	–	–

^a^Avoidable deaths happen by each risk factor to the theoretical minim exposure risk level.

^b^WHO-based scenario10: Avoidable deaths happen by reduce each risk factor to WHO recommended level compared 2010.

^c^WHO-based scenario15: Avoidable deaths happen by reduce each risk factor to WHO recommended level compared 2015.

### WHO-based scenario

**Figure 4:**
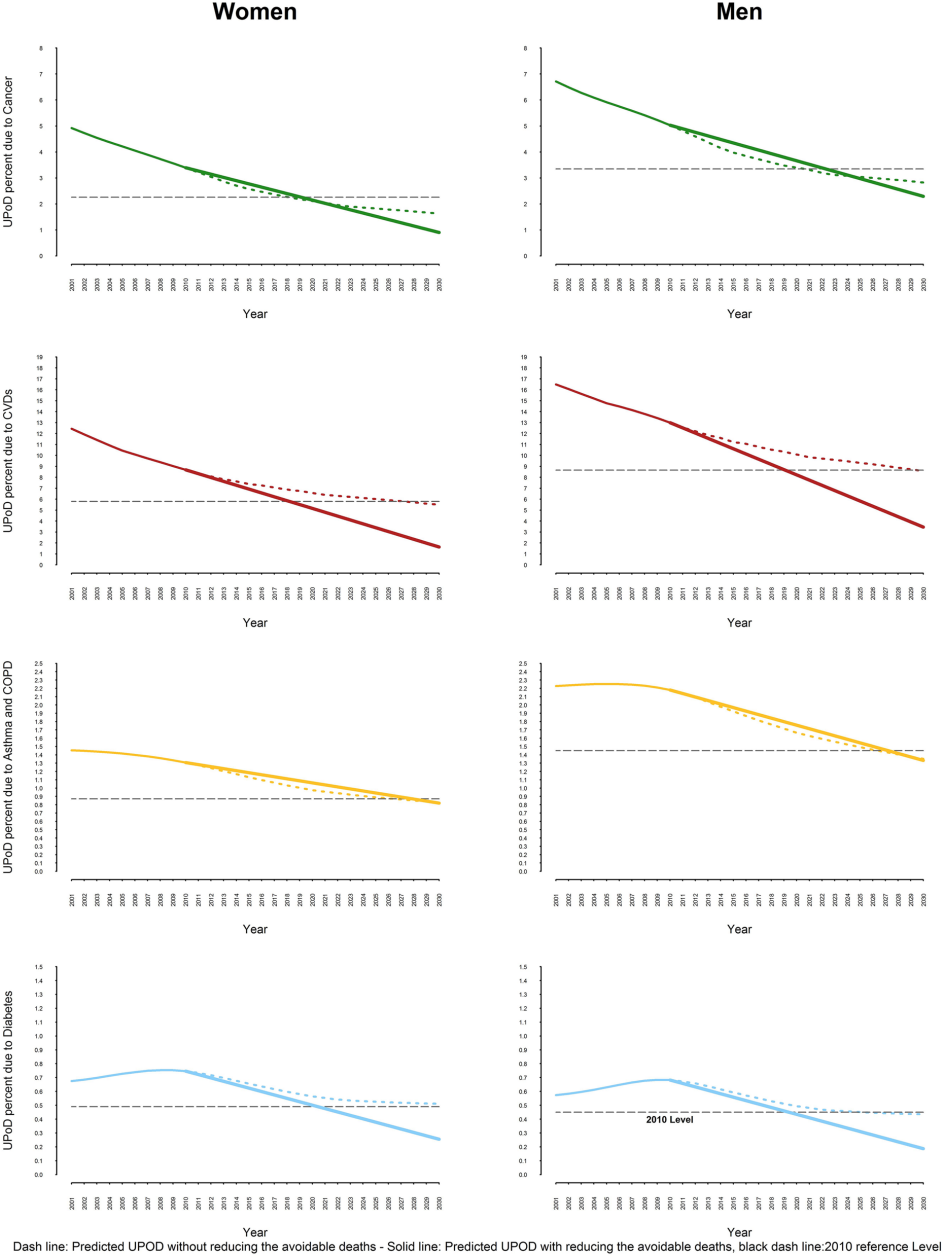
Comparing the projected UPoD (if the current trend goes on) with the UPoD after reducing the avoidable deaths in 2010 WHO-based scenario.

**Figure 5:**
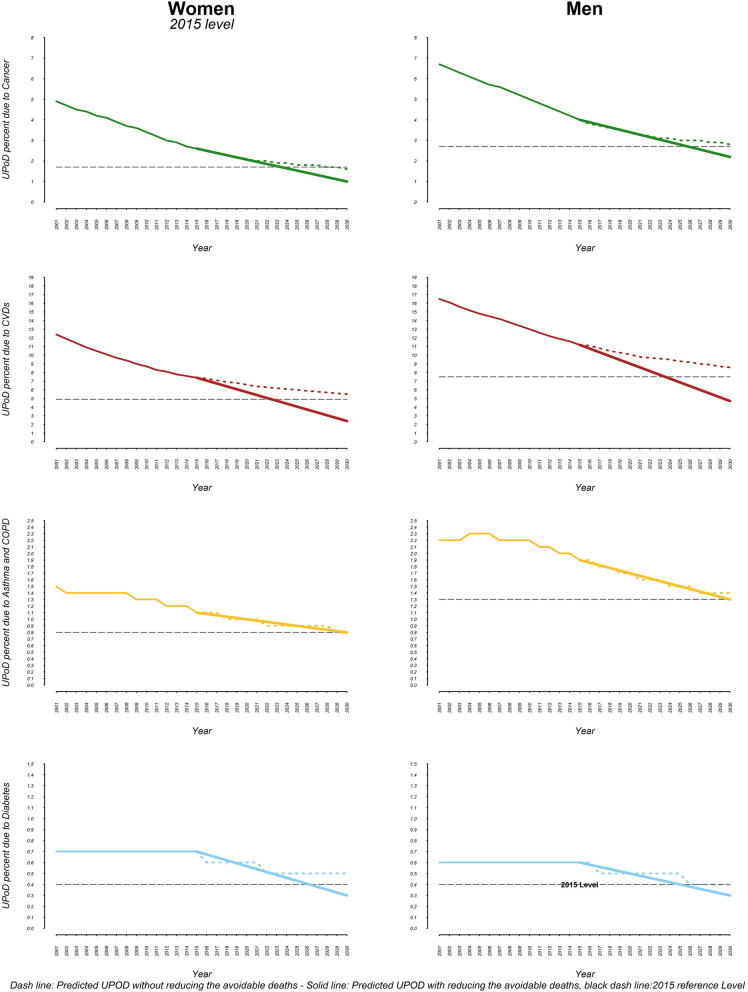
Comparing the projected UPoD (if the current trend goes on) with the UPoD after reducing the avoidable deaths in 2015 WHO-based scenario.

If the risk factor targets determined by WHO are met, Iran will meet SDG 3.4 in both 2010 and 2015 baseline scenarios. Iran, at national level, will achieve the goal for each of the four NCD categories in 2010 baseline scenario (Fig. 4). However the country will not achieve a one-third reduction in asthma and COPD related UPoDs in 2015 baseline scenario (Fig. 5). By 2030, cancer, CVDs, asthma & COPD and diabetes related UPoDs will be respectively 1.59, 2.54, 1.07 and 0.22 in 2010 baseline scenario and 1.60, 3.55, 1.05, and 0.30 in 2015 baseline scenario. The rise in life expectancy is shown in Table 4.

**Table 4:** Gain in life expectancy after elimination of major causes of death from NCDs.

	Gender	Life expectancy			
Life expectancy in 30–70 age group by 2015	Female	39.62			
	Male	36.30			
Life expectancy in 30–70 age group by 2030(projected)	Female	45.46			
	Male	40.63			

		Cancers	CVDs	Asthma and COPD	Diabetes
Gains in life expectancy after elimination in WHO based scenario10	Female	0.18007	1.70329	0.00273	0.05972
	Male	0.17576	2.40042	0.00515	0.06189
Gains in life expectancy after elimination in WHO based scenario15	Female	0.15068	1.18563	0.00245	0.03735
	Male	0.13693	1.47734	0.00466	0.03437

### Subnational

#### Current situation

**Figure 6:**
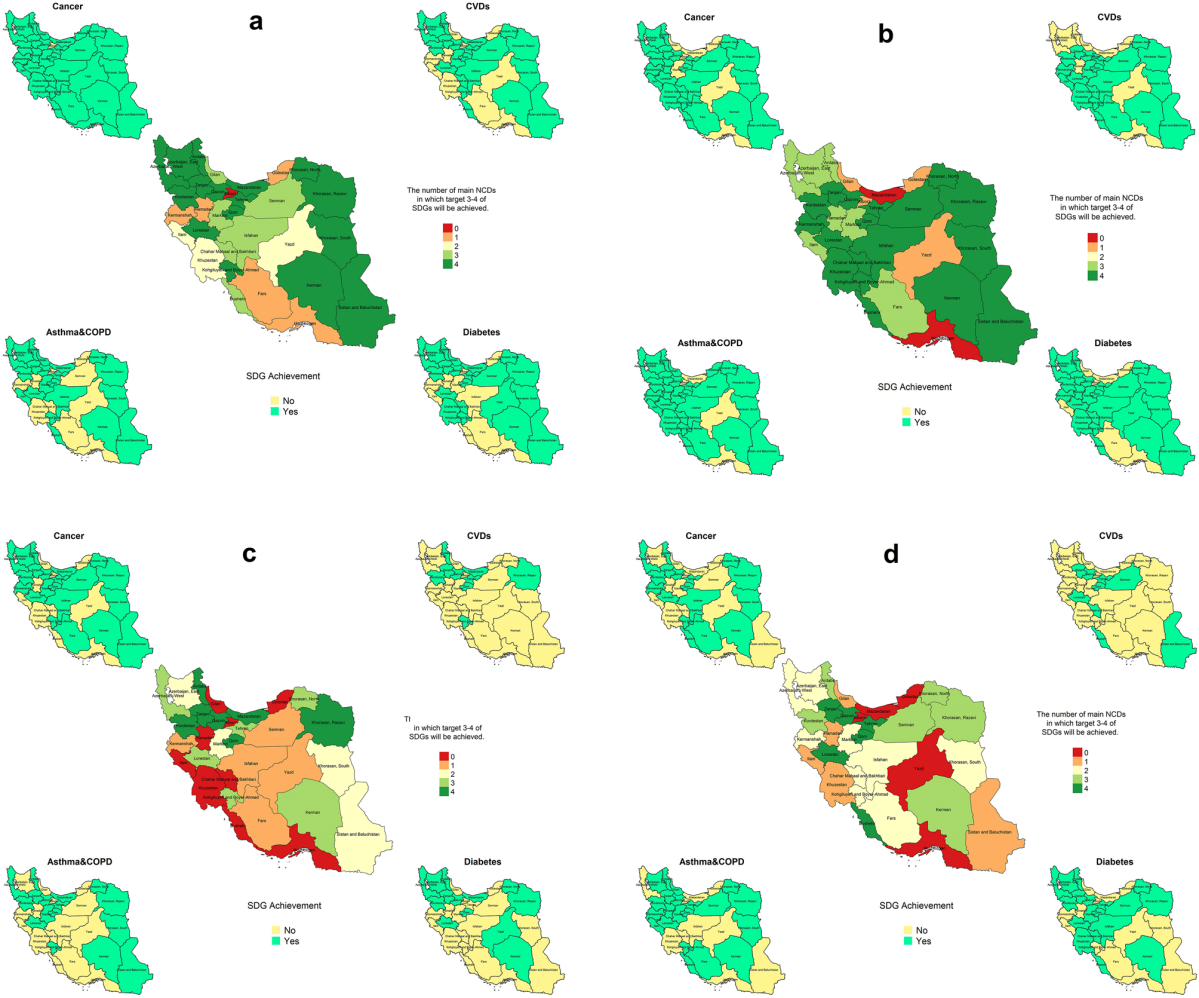
Achieving SDG 3.4 in current situation in the 2 scenarios. (**a**) Female in 2010 baseline scenario, (**b**) male in 2010 baseline scenario, (**c**) female in 2015 baseline scenario, (**d**) male in 2015 baseline scenario. The map was generated in R software version 4.0.1 (2020-04), https://cran.r-project.org/bin/windows/base/.

In 2010 baseline scenario, if the current trend prevails, 30 out of 31 provinces will meet SDG 3.4 among females in cancer by 2030. Also, by the same time-period, 21 provinces will achieve the targets in CVDs, asthma and COPD, and diabetes (Fig. 6a). Plus, 24, 22, 27, and 24 provinces will meet SDG 3.4 in cancer, CVDs, asthma and COPD, and diabetes among males respectively (Fig. 6b).

On the other hand, based on 2015 baseline scenario, by the year 2030, to total number of 21, 9, 16, and 18 provinces will reach the target in cancer, CVDs, asthma and COPD, and diabetes among females respectively (Fig. 6c). Moreover, 17, 9, 21, and 18 provinces will meet SDG 3.4 respectively in cancer, CVDs, asthma and COPD, and diabetes among males (Fig. 6d).

#### WHO-based scenarios

**Figure 7:**
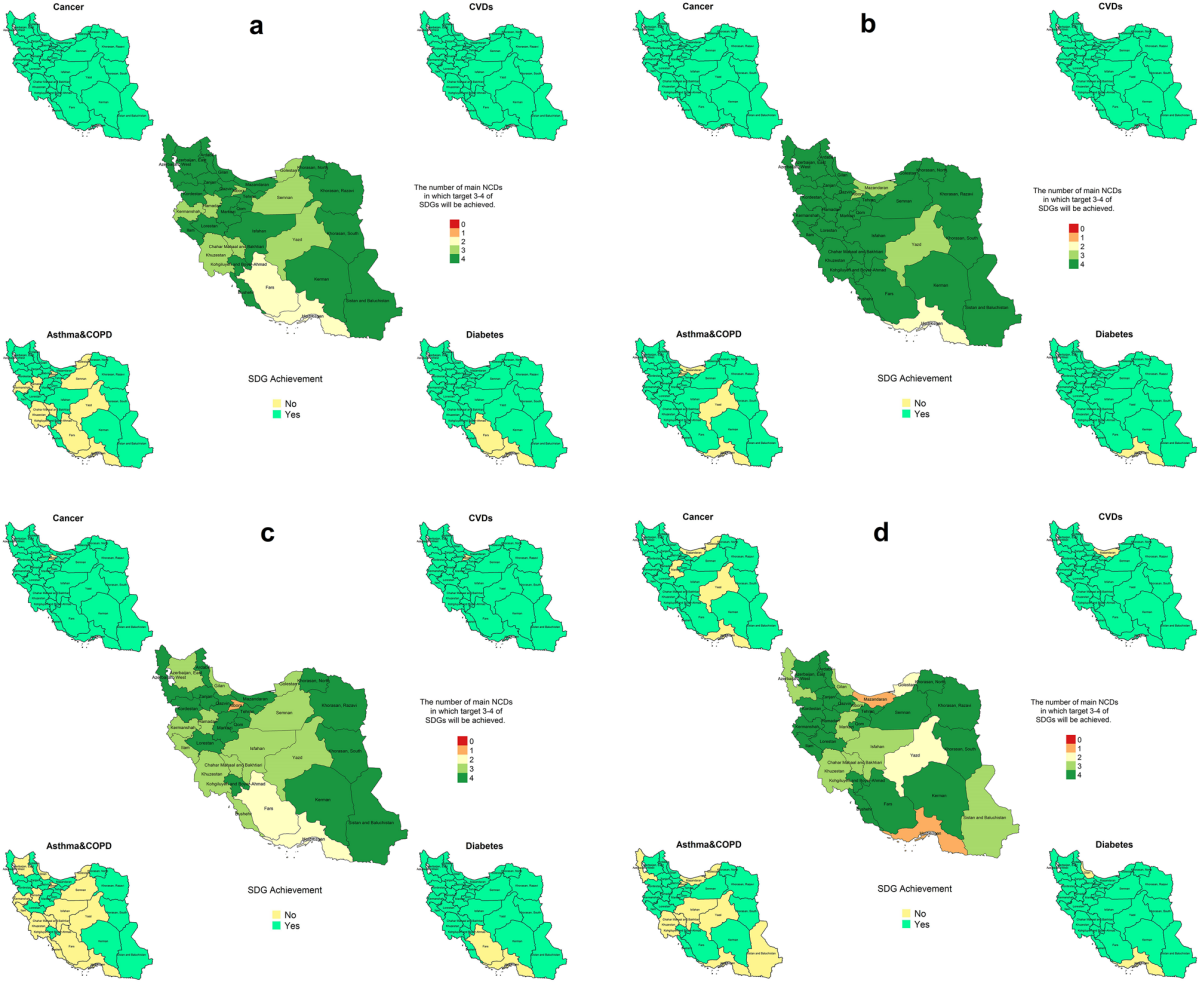
Achieving SDG 3.4 in WHO based scenario in the 2 scenarios. (**a**) Female in 2010 WHO based scenario, (**b**) male in 2010 WHO based scenario, (**c**) female in 2015 WHO based scenario, (**d**) male in 2015 WHO based scenario. The map was generated in R software version 4.0.1 (2020-04), https://cran.r-project.org/bin/windows/base/.

As indicated by the Joint effect of the risk factors in WHO based scenarios, all of the provinces in cancer and CVDs; 21 provinces in asthma and COPD, and 29 provinces in diabetes will achieve SDG 3.4 designed for females. The corresponding number of provinces are 27 and 30 for males (WHO-based scenario10). Also, 30 provinces will reach SDG 3.4 in cancer and CVDs in females; whereas, the number of the provinces meet SDG 3.4 in cancer and CVDs will be 26 and 30 in males respectively. Plus, 15 provinces in asthma and COPD, and 29 province in diabetes will achieve SDG 3.4 in females. On the other hand, 15 ad 29 provinces will reach SDG 3.4 in asthma and COPD and diabetes in males respectively (Fig. 7).

## Discussion

The results showed that with continuation of the current trend (adjusted with the prevalence of risk factors), Iran will not achieve SDG 3.4; thus, in order to achieve a one-third drop in UPoD, some interventions should be conducted in 2015 baseline scenario. On the flip side, the country will be close to achieving the one-third reduction in UPoD (meeting SDG 3.4) in 2010 baseline scenario without any interventions. Obviously, SDG 3.4 wouldn’t be achieved by reducing just one single risk factor. In both 2010 and 2015 baseline scenarios, estimating the joint effect of risk factors could prevent 60.5 and 48% of the projected deaths by 2030, respectively. In 2010 baseline scenario, reducing avoidable deaths will result in achieving SDG 3.4 in Iran; whereas, in 2015 baseline scenario, the country will only achieve SDG 3.4 in cancer, cardiovascular disease, and diabetes; therefore, more efforts and serious interventions are needed in terms of asthma and COPD. It worth mentioning that there is considerable diversity among the provinces regarding the achievement of SDG 3.4.

Iran has been experiencing a downward trend in deaths from non-communicable diseases and this trend will be continued by 2030^16^; however, this downward trend is not enough for achieving SDG 3.4. An international study showed that Iran will meet the target by 2030 in 2015 baseline scenario at national level^17^. The result of the aforementioned study is not consistent with those of our study. The reason behind this inconsistency may be the employed data. In our study, the total of 524,174 real data points were used for modeling. The data was divided by province, age, cause, sex in 15 years; whereas, the results from the other study^17^ were model driven.

Another study conducted in Mexico showed that, in 2010 baseline scenario, this country will not be able to achieve SDG 3.4 if the current situation prevails^18^. The mortality rate from non-communicable diseases in Mexico has declined to 22.9% which it not enough for the nation to achieve SDG 3.4. In our study, in 2010 baseline scenario, relative change of UPoD will be -39% in 2030. That is, the country will meet the target but the achievement will not be equally distributed in all the NCD categories and provinces. Also, Iran will experience a 27% decline in UPoD in 2015 baseline scenario.

Another study concluded that if the current trends in risk factors remain stable in China by 2030, 13.1% of premature deaths from NCDs will be decreased resulting in failure to achieve SDG 3.4. In the combined scenario, in which all the risk factor reduction targets are achieved, the mortality from cardiovascular and COPD will be reduced by one-third^19^. However, Iran will not meet the target in asthma and COPD category.

The present study was carried out using a huge bulk of integrated datasets, providing a projection for the risk factors (for the first time). Based on the current trend and given the targets set by the WHO for risk factors, the present study defined different scenarios and provided the benefits of intervention, or a series of interventions, in light of reducing in the number of deaths. The present study is one of the first few investigations conducted on the issue. It employs a comprehensive approach with modeling and projection to help policy-making and planning for NCDs.

The results of the study provide a brand new dashboard for decision making and optimal allocation of resources by policy makers in the health system. For projection of UPoD, several models were used; in order to employ all possible models and to avoid missing data, BMA was used for projection of UPoD. Another strength of the study is the use of valid RRs extracted from globally significant studies; as a result, the residual confounding was minimized. All analyses were performed by categorization of sex and province. Considering the vast spectrum of this study, there were some limitations; first, the alcohol consumption trend was not available in Iran and due to its low prevalence in 2016 STEPs data (supplement appendix 4), this risk factor was excluded from the study. We made an effort to confront data sacristy challenges with various modeling techniques including Sptio-temporal and GPR. Changes in provincial administrative divisions and increased number of provinces were addressed by Misalignment approach. Also, Alterations in definitions over the time were modified by Cross Walk method and lack of data on the prevalence of risk factors for the age group of 65–69 years in the STEPs before 2011 was addressed by Fractional Polynomial Models.

According to the projections performed in this study, Iran will face a critical situation in which people affected with multiple NCDs risk factors. Hypertension, physical inactivity, obesity, and diabetes are on the rise and despite the decreasing trend in mean of salt intake, this risk factor is, still, higher than the global standard. Therefore, there should be interventions designed and implemented at the individual, population and health system levels.

In this study, hypertension accounts for the highest number of avoidable deaths (this risk factor is prioritized at the interventions). According to the results of STEPs survey performed in 2016, 54% of people in 30–70 year age-group are aware of hypertension, of which 37% receive treatments. Hypertension in 37% of these individuals, is under control^20^. Therefore, the main measures for reducing the hypertension, would be boosting awareness and perusing effective coverage of the controlled hypertension. It is recommended to conduct national preparation program to identify and control hypertension and develop a set of self-care management strategies. Sensitization should be performed up to the extent that each person is aware of their own blood pressure number. Also, part of the hypertension related avoidable deaths is related to the high level of salt intake. Given that about 70% of the salt is sodium and it is added to the food outside the home^21^, it is suggested that intersectoral collaboration interventions for modification in the processes of production of food industry be conducted to reduce salt intake. At the population level, mass media can be employed to change the consumer behaviors; also, launching some strategic campaigns would reinforce the impact of mess media. At the individual level, the "table without shaker" campaign may be adopted as an effective intervention.

The second highest number of avoidable deaths (After hypertension) is associated with physical inactivity and obesity. To reduce avoidable deaths from physical inactivity, infrastructures should be provided at the macro level. Creating an appropriate and safe infrastructures to support hiking and cycling and providing social security to support individuals-especially women-to perform physical activity during their free time, is of significant importance. To increase public awareness and motivate the community to avoid physical inactivity, the mass media can be a game-changer in terms of the behavior at individual and group levels. Sports programs may be designed to be performed in places such as parks, streets, schools and the workplaces. An example of these programs is the ParticipACTION program proposed by the Canadian government to encourage the general population to increase physical activity and to achieve physical health. This program led to considerable reduction in health costs^22^. One of the benefits of a physically active population is the balance of energy and the prevention of obesity^23^. Given the challenges in the implementation of individual interventions to reduce obesity, the emphasis should be put on the population and intersectorial interventions. Apart from interventions designed for increasing physical activity level, it is suggested to educate public on nutrition and counsel them in different environments (such as schools, hospitals, and work environments, …), use food labels to reduce overall energy consumption, propose diet reform, and implement tax on sweet drinks and processed and Fast foods. Including consultation on non-communicable risk factors as part of the Primary Health Services (PHC) would, also, be a cost-effective measure.

## Conclusions

Given the current trends, Iran is unlikely to achieve SDG 3.4. Therefore, it is recommended that the Ministry of Health, besides intersectorial collaboration with the other organizations, consider all life-style and pharmacological interventions at individual and population levels. Inter-sectorial health system interventions appears to produce clear path for public, health care community, policy makers, and insurance companies to achieve SDG 3.4 while there is still time to the end of SDGs deadline.

## Supplementary Information

Supplementary Information.
