# Supplementary material for: Iran to achieve the SDG 3.4 at national and sub-national levels
Source: Sci Rep. 2022 Mar 8;12:3705. doi: 10.1038/s41598-022-07441-8 (PMC8904528; doi:10.1038/s41598-022-07441-8)
Supplement: Supplementary file 1 — Supplementary Information. [file 41598_2022_7441_MOESM1_ESM.docx]

Supplementary

**Appendix 1: Trend and projection of the risk factors**

**Appendix 2: Pairs of the risk factor-disease**

| # | Cause | smoking | Obesity | hypertension | diabetes | salt | Physical inactivity |
| --- | --- | --- | --- | --- | --- | --- | --- |
| 1 | Mouth and oropharynx cancers |  |  |  |  |  |  |
| 2 | Oesophagus cancer |  |  |  |  |  |  |
| 3 | Stomach cancer |  |  |  |  |  |  |
| 4 | Colon and rectum cancers |  |  |  |  |  |  |
| 5 | Liver cancer |  |  |  |  |  |  |
| 6 | Pancreas cancer |  |  |  |  |  |  |
| 7 | Trachea, bronchus and lung cancers |  |  |  |  |  |  |
| 8 | Melanoma and other skin cancers |  |  |  |  |  |  |
| 9 | Breast cancer |  |  |  |  |  |  |
| 10 | Cervix uteri cancer |  |  |  |  |  |  |
| 11 | Corpus uteri cancer |  |  |  |  |  |  |
| 12 | Ovary cancer |  |  |  |  |  |  |
| 13 | Prostate cancer |  |  |  |  |  |  |
| 14 | Bladder cancer |  |  |  |  |  |  |
| 15 | Non-Hodgkin lymphoma |  |  |  |  |  |  |
| 16 | Multiple myeloma |  |  |  |  |  |  |
| 17 | Other lymphomas and multiple myeloma |  |  |  |  |  |  |
| 18 | Leukaemia |  |  |  |  |  |  |
| 19 | Kidney cancer |  |  |  |  |  |  |
| 20 | Gallbladder cancer |  |  |  |  |  |  |
| 21 | Thyroid cancer |  |  |  |  |  |  |
| 22 | Diabetes mellitus |  |  |  |  |  |  |
| 23 | Rheumatic heart disease |  |  |  |  |  |  |
| 25 | Hypertensive heart disease |  |  |  |  |  |  |
| 26 | Ischaemic heart disease |  |  |  |  |  |  |
| 27 | Ischaemic stroke |  |  |  |  |  |  |
| 28 | Haemorrhagic and other non-ischaemic stroke |  |  |  |  |  |  |
| 29 | Cardiomyopathy, myocarditis, endocarditis |  |  |  |  |  |  |
| 30 | Atrial fibrillation and flutter |  |  |  |  |  |  |
| 31 | Other circulatory diseases |  |  |  |  |  |  |
| 32 | Chronic obstructive pulmonary disease |  |  |  |  |  |  |
| 33 | Asthma |  |  |  |  |  |  |
| 34 | Other respiratory diseases |  |  |  |  |  |  |
| 35 | Kidney diseases |  |  |  |  |  |  |

**Appendix 3: Prevalence of risk factors at sub-national level**

| Table 1. The age-standardized prevalence and projection of diabetes in three time periods at subnational level | | | | | | | | | | | | |
| --- | --- | --- | --- | --- | --- | --- | --- | --- | --- | --- | --- | --- |
|  | 2001 | | | | 2016 | | | | 2030 | | | |
|  | Female | | Male | | Female | | Male | | Female | | Male | |
| province | P | 95%CI | P | 95%CI | P | 95%CI | P | 95%CI | P | 95%CI | P | 95%CI |
| Markazi | 7.2 | (6.7- 7.6) | 5.8 | (5.4- 6.3) | 10.0 | (9.6- 10.4) | 8.7 | (8.3- 9.2) | 12.5 | (12.0- 12.9) | 11.1 | (10.7-11.6) |
| Gilan | 7.5 | (7.0- 8.0) | 5.9 | (5.4- 6.3) | 10.1 | (9.7- 10.5) | 8.6 | (8.2- 9.0) | 12.7 | (12.2- 13.1) | 11.2 | (10.8-11.6) |
| Mazandaran | 9.6 | (9.1- 10.0) | 7.8 | (7.4- 8.3) | 11.3 | (10.8- 11.8) | 9.2 | (8.8- 9.7) | 12.8 | (12.2- 13.2) | 10.4 | (9.8-10.9) |
| Azerbaijan_East | 8.2 | (7.8- 8.6) | 6.7 | (6.3- 7.1) | 10.2 | (9.8- 10.6) | 8.6 | (8.2- 9.0) | 12.0 | (11.6- 12.4) | 10.3 | (9.9-10.7) |
| Azerbaijan_West | 7.3 | (6.8- 7.7) | 5.8 | (5.4- 6.2) | 9.8 | (9.3- 9.2) | 8.0 | (7.6- 8.4) | 12.1 | (11.6- 12.5) | 9.7 | (9.3-10.1) |
| Kermanshah | 7.7 | (7.3- 8.1) | 6.2 | (5.8- 6.6) | 10.1 | (9.6- 10.5) | 8.1 | (7.7- 8.5) | 12.2 | (11.7- 12.6) | 9.7 | (9.2-10.1) |
| Khuzestan | 10.1 | (9.7- 10.5) | 8.3 | (7.9- 8.7) | 12.0 | (11.6- 12.4) | 9.9 | (9.5- 10.4) | 13.5 | (13.0- 13.9) | 11.1 | (10.6-11.5) |
| Fars | 8.2 | (7.8- 8.6) | 6.6 | (6.2- 7.0) | 9.9 | (9.5- 9.3) | 8.1 | (7.7- 8.5) | 11.4 | (11.0- 11.8) | 9.3 | (8.9-9.8) |
| Kerman | 6.6 | (6.2- 7.7) | 5.5 | (5.1- 6.0) | 7.7 | (7.2- 7.1) | 6.5 | (6- 6.9) | 8.3 | (7.9- 8.9) | 6.9 | (6.4-7.4) |
| Khorasan_Razavi | 6.4 | (6.1- 6.9) | 5.3 | (4.9- 5.7) | 8.6 | (8.2- 8.9) | 7.1 | (6.7- 7.6) | 10.4 | (10- 10.8) | 8.5 | (8.1-8.9) |
| Isfahan | 9.8 | (9.2- 10.2) | 8.1 | (7.6- 8.6) | 12.0 | (11.5- 12.5) | 10.3 | (9.8- 10.7) | 14.3 | (13.8- 14.8) | 12.3 | (11.8-12.7) |
| Sistan&Baluchistan | 6.3 | (5.8- 6.8) | 5.2 | (4.7- 5.6) | 8.5 | (8- 8.9) | 6.8 | (6.3- 7.3) | 10.4 | (9.9- 10.9) | 8.1 | (7.5-8.6) |
| Kurdistan | 6.3 | (5.9- 6.8) | 5.0 | (4.6- 5.4) | 9.4 | (8.9- 9.9) | 7.4 | (7- 7.8) | 12.1 | (11.6- 12.6) | 9.3 | (8.9-9.8) |
| Hamadan | 6.5 | (6.1- 6.9) | 5.3 | (4.8- 5.7) | 8.6 | (8.2- 8.9) | 6.9 | (6.5- 7.4) | 10.3 | (9.9- 10.7) | 8.2 | (7.7-8.6) |
| ChaharM&Bakhtiari | 6.9 | (6.5- 7.4) | 5.3 | (5.0- 5.8) | 8.4 | (7.9- 8.8) | 7.0 | (6.6- 7.4) | 9.5 | (9.1- 10) | 8.3 | (7.9-8.8) |
| Lorestan | 6.6 | (6.2- 7.1) | 5.3 | (4.9- 5.7) | 8.6 | (8.1- 8.9) | 7.1 | (6.7- 7.5) | 10.3 | (9.9- 10.8) | 8.5 | (8.1-9.0) |
| Ilam | 6.0 | (5.6- 6.5) | 4.7 | (4.2- 5.1) | 8.1 | (7.7- 8.5) | 6.6 | (6.2- 7.0) | 9.9 | (9.4- 10.3) | 8.2 | (7.7-8.6) |
| Kohgiluyeh&BoyerA | 4.9 | (4.5- 5.4) | 3.7 | (3.3- 4.2) | 8.0 | (7.5- 8.4) | 6.3 | (5.8- 6.7) | 10.8 | (10.3- 11.2) | 8.4 | (8-8.9.0) |
| Bushehr | 7.0 | (6.6- 7.4) | 5.3 | (4.9- 5.7) | 8.4 | (8- 8.8) | 7.5 | (7.1- 7.9) | 9.6 | (9.2- 10.1) | 9.9 | (9.4-10.4) |
| Zanjan | 6.2 | (5.8- 6.6) | 4.9 | (4.5- 5.4) | 8.7 | (8.2- 8.1) | 7.4 | (6.9- 7.8) | 10.6 | (10.1- 11.2) | 9.2 | (8.6-9.7) |
| Semnan | 9.6 | (9.1- 10.0) | 8.2 | (7.7- 8.6) | 11.1 | (10.7- 11.6) | 9.8 | (9.4- 10.2) | 12.3 | (11.9- 12.8) | 11.2 | (10.7-11.6) |
| Yazd | 11 | (10.5- 11.4) | 9.2 | (8.8- 9.6) | 12.5 | (12- 12.9) | 10.4 | (9.9- 10.8) | 13.6 | (13.2- 14.1) | 11.1 | (10.6-11.5) |
| Hormozgan | 5.7 | (5.3- 6.2) | 4.6 | (4.1- 5.0) | 7.6 | (7.2- 7.1) | 6.2 | (5.8- 6.7) | 9.3 | (8.8- 9.8) | 7.5 | (7.0- 8.0) |
| Tehran | 9.2 | (8.6- 9.9) | 7.5 | (6.9- 8.1) | 10.5 | (10- 10.11) | 9.1 | (8.6- 9.6) | 11.6 | (11.1- 12.1) | 10.6 | (10.1-11.1) |
| Ardabil | 6.6 | (6.1- 7.7) | 5.2 | (4.7- 5.6) | 9.1 | (8.7- 9.5) | 7.5 | (7.0- 7.9) | 11.1 | (10.7- 11.5) | 9.4 | (8.9-9.8) |
| Qom | 11.6 | (11.0- 12.0) | 9.4 | (8.7- 9.9) | 13.1 | (12.5- 13.7) | 10.8 | (10.3- 11.4) | 14.3 | (13.8- 14.8) | 11.8 | (11.3-12.3) |
| Qazvin | 8.3 | (8.0- 8.8) | 7.0 | (6.6- 7.4) | 10.8 | (10.4- 10.2) | 9.5 | (9.1- 9.9) | 13 | (12.6- 13.4) | 11.7 | (11.2-12.2) |
| Golestan | 7.3 | (6.9- 7.8) | 5.7 | (5.3- 6.2) | 9.1 | (8.6- 9.6) | 7.1 | (6.6- 7.5) | 10.7 | (10.3- 11.2) | 8.1 | (7.6-8.6) |
| Khorasan_North | 5.6 | (5.1- 6.1) | 4.7 | (4.2- 5.1) | 7.7 | (7.3- 7.2) | 6.5 | (6.0- 6.9) | 9.7 | (9.2- 10.2) | 7.9 | (7.4-8.4) |
| Khorasan_South | 5.7 | (5.3- 6.1) | 4.7 | (4.3- 5.2) | 7.7 | (7.3- 7.2) | 6.2 | (5.7- 6.7) | 9.5 | (9.0- 9.9) | 7.4 | (6.9-7.8) |
| Alborz | 10.4 | (9.9- 10.9) | 8.5 | (8.0- 9.1) | 11.7 | (11.2- 11.2) | 10.4 | (9.9- 10.9) | 12.8 | (12.3- 13.3) | 12.3 | (11.7-12.8) |
| Iran | 7.6 | (7.2- 8.1) | 6.2 | (5.7- 6.7) | 9.6 | (9.1- 9.10) | 8 | (7.6- 8.5) | 11.2 | (10.8- 11.7) | 9.5 | (9.0-10) |

| Table 2. The age-standardized prevalence and projection of hypertension in three time periods at sub-national level | | | | | | | | | | | | |
| --- | --- | --- | --- | --- | --- | --- | --- | --- | --- | --- | --- | --- |
|  | 2001 | | | | 2016 | | | | 2030 | | | |
|  | Female | | Male | | Female | | Male | | Female | | Male | |
| province | P | 95%CI | P | 95%CI | P | 95%CI | P | 95%CI | P | 95%CI | P | 95%CI |
| Markazi | 15.5 | (12.6-18.3) | 13.8 | (11.7-15.6) | 29.8 | (27.5-32.0) | 24.2 | (22.4- 25.9) | 42.0 | (38.4- 46.0) | 34.8 | (31.2-38.5) |
| Gilan | 18.8 | (14.4-23) | 14.9 | (12.1-18.4) | 38.5 | (35.5-41.4) | 28.6 | (26.3- 30.8) | 54.1 | (49.8- 58.6) | 41.5 | (37.4-45.7) |
| Mazandaran | 18.3 | (15.1-21.5) | 15.0 | (12.8-18.1) | 34.9 | (32.5-37.3) | 25.7 | (24.0- 27.4) | 47.7 | (44.3- 51.1) | 35.3 | (32.6-38.4) |
| Azerbaijan_East | 19.8 | (16.9-22.6) | 16.5 | (14.2-19.9) | 34.6 | (32.4-36.8) | 27.9 | (26.0- 29.7) | 46.4 | (43.2- 50.0) | 38.5 | (35.2-42.1) |
| Azerbaijan_West | 18.3 | (15.3-21.3) | 15.7 | (13.6-18.3) | 33.6 | (31.3-35.9) | 26.2 | (24.5- 27.9) | 45.9 | (42.5- 49.7) | 35.6 | (32.9-38.6) |
| Kermanshah | 17.9 | (14.5-21.2) | 15.2 | (12.7-17.5) | 34.2 | (31.7-36.7) | 26.8 | (24.8- 28.7) | 47.5 | (43.9- 51.4) | 37.7 | (34.4-41.2) |
| Khuzestan | 16.5 | (13.4-19.5) | 15.1 | (12.9-16.4) | 31.5 | (29.1-33.8) | 25.9 | (24.1- 27.7) | 44.0 | (40.5- 47.9) | 36.2 | (32.7-40.5) |
| Fars | 16.0 | (14.0-18.0) | 13.5 | (11.6-118) | 27.0 | (25.3-28.6) | 23.0 | (21.5- 24.5) | 36.0 | (33.0- 38.9) | 31.6 | (29.1-34.4) |
| Kerman | 16.1 | (14.2-18.1) | 13.5 | (12.1-16.2) | 26.8 | (25.1-28.4) | 20.9 | (19.7- 22.1) | 35.6 | (33.2- 38.2) | 27.7 | (25.7-30.2) |
| Khorasan_Razavi | 16.2 | (13.6-18.7) | 15.2 | (13.1-16.6) | 29.6 | (27.5-31.6) | 26.1 | (24.3- 27.8) | 40.7 | (37.6- 44.0) | 35.8 | (33.0-39.0) |
| Isfahan | 15.5 | (13.1-17.9) | 13.1 | (10.9-15.1) | 28.1 | (26.2-30.0) | 23.8 | (22.0- 25.5) | 38.7 | (35.7- 41.8) | 33.5 | (30.7-36.5) |
| Sistan&Baluchistan | 12.1 | (10-14.2) | 11.2 | (9.6-12.14) | 22.5 | (20.7-24.2) | 19.3 | (17.9- 20.7) | 32.2 | (29.2- 35.7) | 27.1 | (24.8-29.7) |
| Kurdistan | 16.2 | (12.5-19.8) | 14.3 | (11.7-16.5) | 33.6 | (30.9-36.2) | 26.6 | (24.5- 28.6) | 48.1 | (44.0- 52.3) | 38.0 | (34.6-41.5) |
| Hamadan | 17.6 | (14.2-21) | 14.7 | (12.7-17.2) | 34.8 | (32.3-37.4) | 24.6 | (23.0- 26.2) | 48.3 | (44.6- 52.2) | 33.8 | (31.0-36.9) |
| ChaharM&Bakhtiari | 19.0 | (15.9-22.1) | 16.9 | (14.9-19.0) | 34.7 | (32.3-37.1) | 27.5 | (25.7- 29.1) | 47.4 | (43.9- 51.0) | 36.9 | (34.0-40.3) |
| Lorestan | 16.9 | (12.9-20.7) | 15.2 | (13.0-16.9) | 33.9 | (31.1-36.6) | 25.3 | (23.5- 27.0) | 48.3 | (43.9- 53.0) | 35.3 | (32.0-39.0) |
| Ilam | 13.3 | (9.5-16.9) | 12.3 | (10.1-13.5) | 29.9 | (27.1-32.5) | 23.2 | (21.3- 25.0) | 44.8 | (40.0- 49.4) | 33.3 | (30.0-36.9) |
| Kohgiluyeh&BoyerA | 17.2 | (14.2-20.1) | 14.5 | (12.3-17.2) | 31.6 | (29.4-33.9) | 25.1 | (23.3- 26.8) | 43.9 | (40.5- 47.6) | 35.2 | (32.0-38.7) |
| Bushehr | 18.6 | (16.4-20.7) | 15.3 | (13.7-18.4) | 30.2 | (28.4-32.0) | 23.3 | (22.0- 24.7) | 39.2 | (36.0- 42.0) | 30.8 | (28.5-33.3) |
| Zanjan | 17.9 | (15.1-20.7) | 15.5 | (13.7-17.1) | 32.6 | (30.4-34.8) | 25.1 | (23.6- 26.6) | 44.3 | (41.0- 47.7) | 33.6 | (31.1-36.5) |
| Semnan | 17.0 | (14.1-19.9) | 15.5 | (13.1-11.0) | 31.7 | (29.4-33.9) | 27.6 | (25.6- 29.5) | 43.5 | (40.3- 46.9) | 38.2 | (35.0-41.8) |
| Yazd | 13.7 | (11.7-15.7) | 11.4 | (9.6-13.7) | 24.6 | (22.9-26.3) | 20.6 | (19.1- 22.0) | 33.6 | (30.9- 36.2) | 29.1 | (26.5-31.9) |
| Hormozgan | 14.9 | (12.5-17.4) | 13.4 | (11.2-14.5) | 27.9 | (25.9-29.8) | 24.2 | (22.4- 25.9) | 38.5 | (35.6- 41.6) | 34.0 | (30.9-37.4) |
| Tehran | 15.6 | (13.6-17.7) | 14.8 | (12.6-15.6) | 26.4 | (24.6-28.1) | 25.8 | (24.0- 27.6) | 35.5 | (32.5- 38.6) | 35.8 | (32.8-39.1) |
| Ardabil | 18.3 | (14.9-21.7) | 16.2 | (13.6-18.9) | 35.0 | (32.5-37.5) | 27.9 | (25.8- 29.8) | 48.6 | (44.7- 52.7) | 40.0 | (36.1-43.9) |
| Qom | 15.5 | (12.8-18.1) | 15.0 | (12.5-15.8) | 28.9 | (26.8-31.0) | 26.8 | (24.8- 28.7) | 40.2 | (37.0- 43.8) | 37.9 | (34.4-41.6) |
| Qazvin | 15.3 | (12.7-17.9) | 14.0 | (12.0-15.7) | 28.9 | (26.8-30.9) | 23.9 | (22.2- 25.5) | 40.1 | (36.9- 43.6) | 33.3 | (30.3-36.5) |
| Golestan | 15.6 | (12.6-18.6) | 12.5 | (10.2-15.6) | 30.3 | (27.9-32.6) | 23.2 | (21.3- 25.0) | 43.0 | (39.2- 47.1) | 34.7 | (30.9-38.2) |
| Khorasan_North | 19.3 | (15.9-22.7) | 17.2 | (14.7-19.9) | 36.6 | (33.9-39.1) | 29.8 | (27.8- 31.8) | 50.1 | (46.1- 54.3) | 40.8 | (37.5-44.6) |
| Khorasan_South | 15.2 | (12.2-18.1) | 14.0 | (12.1-15.2) | 29.0 | (26.7-31.2) | 23.7 | (22.1- 25.3) | 41.4 | (37.8- 45.2) | 32.6 | (30.1-35.1) |
| Alborz | 24.7 | (22.6-26.7) | 22.4 | (20.4-24.6) | 34.9 | (33.3-36.6) | 32.2 | (30.6- 33.8) | 40.2 | (36.7- 43.7) | 38.5 | (34.7-41.8) |
| Iran | 16.9 | (14.1-19.7) | 15.0 | (12.9-16.1) | 30.9 | (28.7-33.1) | 25.5 | (23.8- 27.2) | 42.5 | (39- 46.1) | 35.2 | (32.1-38.5) |

| Table 3. The age-standardized prevalence and projection of obesity and overweight in three time periods at sub-national level | | | | | | | | | | | | |
| --- | --- | --- | --- | --- | --- | --- | --- | --- | --- | --- | --- | --- |
|  | 2001 | | | | 2016 | | | | 2030 | | | |
|  | Female | | Male | | Female | | Male | | Female | | Male | |
| province | P | 95%CI | P | 95%CI | P | 95%CI | P | 95%CI | P | 95%CI | P | 95%CI |
| Markazi | 18.2 | (14.1- 22.0) | 9.6 | (7.4-11.6) | 34.9 | (32.7- 37.0) | 19.3 | (17.7-20.8) | 44.9 | (42.3-47.5) | 27.9 | (25.2- 30.8) |
| Gilan | 20.3 | (16.2- 23.9) | 10.9 | (8.8-13) | 36.6 | (34.4- 38.5) | 20.9 | (19.3-22.4) | 45.9 | (43.7-48.2) | 29.4 | (26.7- 32.1) |
| Mazandaran | 23.0 | (18.7- 26.9) | 13.1 | (10.7-15.4) | 40.0 | (37.9- 41.9) | 23.9 | (22.3-25.5) | 49.0 | (46.9-50.9) | 32.4 | (30.0- 34.9) |
| Azerbaijan_East | 19.6 | (15.5- 23.3) | 10.7 | (8.5-12.8) | 36.1 | (33.9- 38.1) | 20.5 | (18.9-22.0) | 45.7 | (43.2-48.2) | 28.9 | (26.4- 31.5) |
| Azerbaijan_West | 20.3 | (15.8- 24.3) | 10.9 | (8.5-13.3) | 37.5 | (35.3- 39.6) | 21.6 | (19.9-23.2) | 47.2 | (44.8-49.7) | 30.2 | (27.7- 33.0) |
| Kermanshah | 18.4 | (14.6- 22.0) | 9.9 | (7.7-11.9) | 34.5 | (32.4- 36.4) | 19.4 | (17.9-20.9) | 43.9 | (41.6-46.2) | 27.6 | (25.2- 30.1) |
| Khuzestan | 18.5 | (14.6- 22.1) | 9.9 | (7.8-11.9) | 34.4 | (32.3- 36.4) | 19.3 | (17.7-20.8) | 44.0 | (41.6-46.5) | 27.7 | (24.9- 31.1) |
| Fars | 15.0 | (11.7- 18.2) | 7.8 | (6.1-9.3) | 29.5 | (27.4- 31.4) | 15.2 | (14.0-16.4) | 39.2 | (36.3-42.5) | 22.0 | (19.9- 24.5) |
| Kerman | 12.9 | (9.9- 15.8) | 6.4 | (4.9-7.9) | 26.1 | (24.2- 28.0) | 13.1 | (12.0-14.3) | 35.6 | (33.1-38.4) | 19.8 | (17.5- 22.1) |
| Khorasan_Razavi | 15.7 | (12.0- 19.2) | 8.1 | (6.2-9.9) | 30.9 | (28.7- 32.9) | 16.6 | (15.2-18.0) | 41.0 | (38.2-43.9) | 24.5 | (22.1- 27.0) |
| Isfahan | 18.3 | (14.5- 21.7) | 9.6 | (7.5-11.6) | 33.7 | (31.6- 35.6) | 18.8 | (17.3-20.2) | 43.2 | (40.8-45.8) | 26.9 | (24.5- 29.5) |
| Sistan&Baluchistan | 10.7 | (8.0- 13.3) | 5.1 | (3.7-6.4) | 22.5 | (20.6- 24.2) | 11.0 | (9.9-12.0) | 31.9 | (29.1-34.7) | 17.0 | (15.1- 19.0) |
| Kurdistan | 17.5 | (13.4- 21.3) | 9.1 | (7.1-11.1) | 34.0 | (31.8- 36.1) | 18.4 | (16.9-19.8) | 44.1 | (41.5-46.6) | 26.3 | (24.0- 28.6) |
| Hamadan | 16.6 | (12.7- 20.2) | 8.8 | (6.9-10.7) | 32.7 | (30.5- 34.7) | 17.5 | (16.1-18.8) | 42.7 | (40.1-45.4) | 25.2 | (22.9- 27.7) |
| ChaharM&Bakhtiari | 16.9 | (13.1- 20.4) | 8.7 | (6.7-10.6) | 32.4 | (30.3- 34.4) | 17.6 | (16.2-19) | 42.3 | (39.7-44.9) | 25.6 | (23.0- 28.3) |
| Lorestan | 16.3 | (12.3- 20.0) | 8.6 | (6.6-10.6) | 32.4 | (30.2- 34.4) | 17.6 | (16.1-19) | 42.2 | (39.7-44.7) | 25.7 | (23.2- 28.5) |
| Ilam | 14.3 | (10.9- 17.5) | 7.5 | (5.8-9.1) | 28.7 | (26.6- 30.6) | 15.1 | (13.8-16.3) | 38.3 | (35.8-40.9) | 22.2 | (19.8- 24.6) |
| Kohgiluyeh&BoyerA | 18.0 | (14.1- 21.6) | 9.3 | (7.2-11.3) | 34.2 | (32.1- 36.2) | 19.1 | (17.6-20.6) | 43.8 | (41.5-46.2) | 27.3 | (24.8- 30.0) |
| Bushehr | 15.8 | (12.4- 18.9) | 8.1 | (6.4-9.8) | 30.3 | (28.2- 32.2) | 16.2 | (14.9-17.5) | 39.9 | (37.3-42.6) | 23.7 | (21.3- 26.0) |
| Zanjan | 17.6 | (13.5- 21.3) | 9.3 | (7.3-11.3) | 34.0 | (31.8- 36.1) | 18.7 | (17.2-20.2) | 44.0 | (41.4-46.7) | 27.1 | (24.4- 29.8) |
| Semnan | 19.0 | (15.2- 22.6) | 9.8 | (7.7-11.9) | 34.8 | (32.7- 36.8) | 19.5 | (18.0-21.0) | 44.4 | (42.0-46.8) | 27.9 | (25.3- 30.8) |
| Yazd | 18.5 | (14.8- 21.9) | 10.0 | (7.9-11.9) | 34.0 | (31.9- 35.9) | 19.0 | (17.5-20.4) | 43.4 | (41.1-45.9) | 27.0 | (24.5- 29.5) |
| Hormozgan | 13.0 | (10.3- 15.5) | 6.2 | (4.8-7.6) | 25.5 | (23.7- 27.1) | 12.9 | (11.8-14.0) | 34.2 | (31.9-36.6) | 19.2 | (17.2- 21.5) |
| Tehran | 20.7 | (17.1- 23.9) | 11.5 | (9.5-13.4) | 36.1 | (34.1- 37.9) | 20.7 | (19.2-22.1) | 45.1 | (42.7-47.6) | 28.5 | (26.2- 31.0) |
| Ardabil | 22.0 | (18.0- 25.6) | 12.2 | (9.7-14.5) | 38.2 | (36.2- 40.1) | 22.9 | (21.2-24.5) | 47.3 | (45.1-49.6) | 31.7 | (29.1- 34.3) |
| Qom | 20.4 | (16.3- 24.2) | 11.2 | (8.8-13.5) | 37.0 | (34.8- 38.9) | 21.7 | (20.0-23.3) | 46.5 | (44.1-48.9) | 30.6 | (27.9- 33.4) |
| Qazvin | 18.6 | (14.6- 22.2) | 9.8 | (7.7-11.8) | 34.7 | (32.5- 36.7) | 19.4 | (17.8-20.9) | 44.6 | (42.0-47.3) | 28.0 | (25.2- 30.7) |
| Golestan | 18.9 | (14.2- 23.1) | 11.9 | (9.6-14) | 36.9 | (34.5- 39.0) | 21.9 | (20.3-23.4) | 47.0 | (44.4-49.5) | 30.4 | (27.9- 33.0) |
| Khorasan_North | 16.1 | (12.6- 19.3) | 7.7 | (5.8-9.7) | 30.8 | (28.7- 32.8) | 16.9 | (15.4-18.3) | 40.8 | (38.0-43.7) | 25.2 | (22.5- 27.9) |
| Khorasan_South | 13.1 | (9.8- 16.0) | 6.2 | (4.5-7.7) | 25.9 | (23.9- 27.8) | 13.2 | (12.0-14.4) | 35.8 | (33.0-38.5) | 20.0 | (18.0- 21.9) |
| Alborz | 20.8 | (17.3- 24.0) | 10.6 | (8.3-12.7) | 35.5 | (33.6- 37.3) | 20.9 | (19.3-22.4) | 44.4 | (42.2-46.7) | 29.4 | (26.9- 32.1) |
| Iran | 17.6 | (13.8- 21.0) | 9.3 | (7.3-11.2) | 33.1 | (31.0- 35.0) | 18.3 | (16.9-19.8) | 42.6 | (40.2-45.2) | 26.3 | (23.8- 28.9) |

**Table 4. The age-standardized prevalence and projection of physical inactivity in three time periods at sub-national level**

|  | 2001 | | | | 2016 | | | | 2030 | | | |
| --- | --- | --- | --- | --- | --- | --- | --- | --- | --- | --- | --- | --- |
|  | Female | | Male | | Female | | Male | | Female | | Male | |
| province | P | 95%CI | P | 95%CI | P | 95%CI | P | 95%CI | P | 95%CI | P | 95%CI |
| Markazi | 29.7 | (24.0- 35.1) | 17.7 | (12.3- 22.8) | 54.8 | (51.3- 58.0) | 43.0 | (39.4- 46.5) | 71.0 | (66.8- 75.4) | 60.5 | (55.8- 65.5) |
| Gilan | 30.2 | (24.4- 35.6) | 18.3 | (12.9- 23.4) | 55.4 | (51.9- 58.6) | 43.6 | (40.0- 47.0) | 71.4 | (67.5- 75.5) | 60.9 | (56.3- 65.7) |
| Mazandaran | 36.2 | (30.2- 41.9) | 24.4 | (18.7- 29.7) | 61.6 | (58.1- 64.7) | 49.8 | (46.2- 53.1) | 76.9 | (73.2- 80.7) | 66.6 | (62.3- 71.0) |
| Azerbaijan_East | 43.6 | (37.2- 49.5) | 31.7 | (25.9- 37.2) | 68.8 | (65.5- 71.9) | 57.0 | (53.5- 60.3) | 83.2 | (79.5- 86.9) | 73.0 | (69.0- 77.2) |
| Azerbaijan_West | 24.5 | (18.8- 29.9) | 12.7 | (7.3- 17.8) | 49.8 | (46.3- 53.2) | 37.9 | (34.3- 41.4) | 66.7 | (62.3- 71.4) | 55.6 | (51.1- 60.2) |
| Kermanshah | 28.6 | (22.8- 34.1) | 16.6 | (11.1- 21.9) | 53.7 | (50.2- 57) | 42.0 | (38.4- 45.4) | 70.0 | (65.9- 74.1) | 59.4 | (55.0- 64.0) |
| Khuzestan | 32.1 | (26.2- 37.5) | 20.1 | (14.6- 25.3) | 57.3 | (53.8- 60.5) | 45.6 | (42.0- 49.0) | 73.1 | (69.2- 77.2) | 63.1 | (58.1- 68.8) |
| Fars | 15.9 | (10.5- 21.1) | 8.3 | (3.0- 13.3) | 41.4 | (37.7- 44.8) | 29.8 | (26.1- 33.2) | 58.8 | (53.7- 64.1) | 48.6 | (42.7- 54.5) |
| Kerman | 26.0 | (20.2- 31.4) | 14.1 | (8.6- 19.4) | 51.4 | (47.9- 54.7) | 39.5 | (35.9- 43.0) | 68.0 | (63.9- 72.3) | 57.1 | (52.8- 61.6) |
| Khorasan_Razavi | 22.8 | (17.2- 28.1) | 10.9 | (5.6- 16.0) | 48.0 | (44.4- 51.3) | 36.2 | (32.6- 39.7) | 64.9 | (60.5- 69.4) | 54.1 | (49.5- 58.9) |
| Isfahan | 40.4 | (34.2- 46.2) | 28.5 | (22.7- 34.0) | 65.5 | (62.1- 68.6) | 53.8 | (50.3- 57.0) | 80.3 | (76.6- 83.9) | 70.1 | (66.0- 74.2) |
| Sistan&Baluchistan | 29.3 | (23.6- 34.8) | 17.4 | (11.9- 22.7) | 54.6 | (51.1- 57.9) | 42.8 | (39.2- 46.2) | 70.8 | (66.7- 74.9) | 60.0 | (55.8- 64.3) |
| Kurdistan | 25.8 | (20.1- 31.1) | 13.9 | (8.6- 19.0) | 51.0 | (47.5- 54.4) | 39.3 | (35.7- 42.7) | 67.7 | (63.5- 71.8) | 56.9 | (52.5- 61.4) |
| Hamadan | 39.7 | (33.5- 45.5) | 27.8 | (22.1- 33.3) | 64.9 | (61.5- 68.1) | 53.2 | (49.6- 56.5) | 80.0 | (76.2- 83.9) | 69.6 | (65.5- 74.0) |
| ChaharM&Bakhtiari | 41.6 | (35.3- 47.5) | 29.5 | (23.8- 35.0) | 66.9 | (63.5- 70.0) | 55.1 | (51.6- 58.4) | 81.6 | (78.0- 85.1) | 71.5 | (67.1- 76.2) |
| Lorestan | 41.5 | (35.0- 47.5) | 29.8 | (23.9- 35.3) | 66.8 | (63.5- 69.9) | 55.2 | (51.6- 58.4) | 81.2 | (77.7- 84.6) | 71.4 | (67.3- 75.6) |
| Ilam | 33.3 | (27.3- 38.9) | 21.5 | (15.9- 26.8) | 58.7 | (55.2- 61.9) | 46.9 | (43.3- 50.3) | 74.4 | (70.5- 78.4) | 63.9 | (59.4- 68.6) |
| Kohgiluyeh&BoyerA | 37.7 | (31.6- 43.3) | 25.7 | (20.1- 31.0) | 62.8 | (59.3- 65.9) | 51.0 | (47.5- 54.4) | 77.9 | (74.2- 81.7) | 67.7 | (63.4- 72.3) |
| Bushehr | 43.1 | (36.7- 49.0) | 31.2 | (25.4- 36.6) | 68.3 | (64.9- 71.4) | 56.5 | (53.0- 59.7) | 82.8 | (79.1- 86.5) | 72.6 | (68.4- 76.9) |
| Zanjan | 37.0 | (30.9- 42.7) | 25.1 | (19.5- 30.4) | 62.3 | (58.8- 65.5) | 50.4 | (46.9- 53.8) | 77.9 | (73.8- 82.0) | 67.2 | (62.8- 71.8) |
| Semnan | 33.8 | (27.9- 39.4) | 21.7 | (16.2- 26.9) | 59.0 | (55.5- 62.2) | 47.2 | (43.6- 50.6) | 74.8 | (70.8- 78.8) | 64.3 | (59.7- 69.3) |
| Yazd | 27.8 | (22.1- 33.3) | 15.9 | (10.4- 21.1) | 53.1 | (49.5- 56.4) | 41.3 | (37.7- 44.7) | 69.5 | (65.4- 73.8) | 58.9 | (54.3- 63.6) |
| Hormozgan | 24.8 | (19.2- 30.1) | 12.7 | (7.4- 17.8) | 50.0 | (46.5- 53.4) | 38.3 | (34.6- 41.7) | 66.8 | (62.5- 71.2) | 56.2 | (51.2- 61.5) |
| Tehran | 34.7 | (28.8- 40.2) | 23.0 | (17.5- 28.3) | 60.3 | (56.8- 63.5) | 48.4 | (44.8- 51.7) | 76.1 | (71.8- 80.9) | 65.3 | (60.9- 69.8) |
| Ardabil | 27.0 | (21.4- 32.4) | 15.2 | (9.8- 20.4) | 52.4 | (48.8- 55.7) | 40.5 | (36.9- 43.9) | 68.9 | (64.6- 73.4) | 58.0 | (53.5- 62.8) |
| Qom | 32.6 | (26.7- 38.2) | 20.8 | (15.2- 26.1) | 58.1 | (54.6- 61.3) | 46.3 | (42.7- 49.7) | 74.1 | (70.0- 78.5) | 63.5 | (59.0- 68.2) |
| Qazvin | 30.7 | (25.0- 36.2) | 18.9 | (13.4- 24) | 55.9 | (52.4- 59.2) | 44.2 | (40.6- 47.6) | 72.1 | (67.9- 76.4) | 61.5 | (56.9- 66.3) |
| Golestan | 27.3 | (21.6- 32.7) | 15.4 | (9.9- 20.7) | 52.7 | (49.2- 56.0) | 41.0 | (37.4- 44.5) | 69.3 | (65.0- 73.8) | 58.8 | (54.1- 63.6) |
| Khorasan_North | 56.4 | (48.6- 63.1) | 44.4 | (37.9- 50.4) | 81.9 | (78.8- 84.6) | 70.0 | (66.7- 73.1) | 93.4 | (90.5- 95.6) | 84.4 | (80.7- 87.9) |
| Khorasan_South | 41.7 | (35.3- 47.6) | 29.7 | (23.9- 35.2) | 66.8 | (63.5- 69.9) | 55.0 | (51.6- 58.3) | 81.4 | (77.9- 84.6) | 71.1 | (67.3- 74.7) |
| Alborz | 41.4 | (35.2- 47.2) | 29.6 | (23.9- 35.0) | 66.7 | (63.3- 69.8) | 54.8 | (51.3- 58.1) | 81.4 | (77.8- 84.9) | 71.1 | (66.9- 75.6) |
| Iran | 33.5 | (27.5- 39.1) | 21.7 | (16.1- 27.0) | 58.7 | (55.3- 61.9) | 47.0 | (43.4- 50.3) | 74.4 | (70.4- 78.4) | 64.0 | (59.5- 68.6) |

| Table 5. The age-standardized prevalence and projection of mean of salt intake in three time periods at sub-national level | | | | | | | | | | | | |
| --- | --- | --- | --- | --- | --- | --- | --- | --- | --- | --- | --- | --- |
|  | 2001 | | | | 2016 | | | | 2030 | | | |
|  | Female | | Male | | Female | | Male | | Female | | Male | |
| province | P | 95%CI | P | 95%CI | P | 95%CI | P | 95%CI | P | 95%CI | P | 95%CI |
| Markazi | 9.9 | (9.9- 10) | 11.9 | (11.6-12.3) | 9.7 | (9.7- 9.8) | 10.2 | (9.9- 10.5) | 9.2 | (9.0- 9.4) | 8.0 | (7.2-8.8) |
| Gilan | 8.6 | (8.5- 8.7) | 10.3 | (10.2-10.4) | 9.1 | (9.0- 9.2) | 9.6 | (9.5- 9.7) | 9.6 | (9.5- 9.8) | 8.5 | (8.2- 8.8) |
| Mazandaran | 10.7 | (10.3- 11.1) | 12.5 | (11.8-13.1) | 9.0 | (8.6- 9.3) | 9.5 | (8.9- 10.1) | 5.9 | (5.0- 6.8) | 4.3 | (2.8- 5.8) |
| Azerbaijan_East | 9.5 | (9.3- 9.7) | 11.5 | (11-11.9) | 8.9 | (8.7- 9.1) | 9.6 | (9.2- 10.0) | 6.6 | (5.9- 7.2) | 5.9 | (4.9- 6.9) |
| Azerbaijan_West | 11.7 | (11.2- 12.2) | 12.8 | (12.2-13.4) | 9.4 | (9.0- 9.9) | 9.8 | (9.3- 10.4) | 5.6 | (4.3- 6.8) | 5.4 | (3.9- 6.9) |
| Kermanshah | 9.7 | (9.5- 9.8) | 9.6 | (9.5-9.8) | 10.5 | (10.4- 10.7) | 10.5 | (10.4- 10.7) | 11.2 | (10.9- 11.5) | 11.2 | (10.9- 11.5) |
| Khuzestan | 9.2 | (9.1- 9.2) | 10.6 | (10.4-10.7) | 9.2 | (9.1- 9.2) | 9.7 | (9.6- 9.9) | 8.7 | (8.6- 8.8) | 8.1 | (7.5- 8.7) |
| Fars | 9.0 | (9.0- 9.1) | 10.6 | (10.4-10.8) | 9.3 | (9.2- 9.4) | 9.9 | (9.7- 10.1) | 8.8 | (8.6- 9.1) | 8.2 | (7.6- 8.7) |
| Kerman | 10.8 | (10.3- 11.3) | 12.0 | (11.4-12.6) | 8.8 | (8.3- 9.2) | 9.2 | (8.6- 9.8) | 4.5 | (3.2- 5.7) | 3.7 | (2.2- 5.2) |
| Khorasan_Razavi | 10.2 | (10.1- 10.4) | 10.0 | (10.0-10.1) | 9.6 | (9.5- 9.8) | 9.7 | (9.6- 9.7) | 8.3 | (7.9- 8.7) | 9.3 | (9.2- 9.5) |
| Isfahan | 9.5 | (9.4- 9.7) | 10.3 | (10.2-10.5) | 9.5 | (9.4- 9.6) | 10.1 | (9.9- 10.2) | 7.7 | (7.2- 8.2) | 8.1 | (7.6- 8.6) |
| Sistan&Baluchistan | 10.0 | (9.8- 10.3) | 11.2 | (10.9-11.6) | 9.0 | (8.8- 9.2) | 9.7 | (9.4- 10.0) | 7.5 | (7.1- 8.8) | 7.7 | (7- 8.3) |
| Kurdistan | 11.2 | (10.9- 11.5) | 10.7 | (10.5-10.9) | 10.2 | (10- 10.5) | 10.4 | (10.2- 10.6) | 8.0 | (7.4- 8.6) | 8.5 | (7.9- 9) |
| Hamadan | 11.1 | (10.8- 11.4) | 12.5 | (12.0-13.0) | 9.9 | (9.6- 10.1) | 10.1 | (9.6- 10.6) | 7.3 | (6.4- 8.8) | 6.3 | (5.1- 7.5) |
| ChaharM&Bakhtiari | 9.8 | (9.7- 10.0) | 10.4 | (10.2-10.7) | 10.0 | (9.9- 10.1) | 9.9 | (9.7- 10.1) | 8.8 | (8.4- 9.2) | 7.3 | (6.7- 7.9) |
| Lorestan | 10.5 | (10.3- 10.8) | 11.5 | (11.2-11.8) | 9.6 | (9.3- 9.8) | 10.2 | (9.9- 10.5) | 7.2 | (6.3- 8.8) | 7.5 | (6.7- 8.3) |
| Ilam | 10.4 | (10.1- 10.6) | 11.3 | (10.9-11.7) | 9.1 | (8.9- 9.4) | 9.4 | (9.0- 9.7) | 7.0 | (6.4- 7.5) | 7.2 | (6.4- 8.0) |
| Kohgiluyeh&BoyerA | 11.1 | (10.7- 11.5) | 12.5 | (11.9-13.1) | 9.3 | (9.0- 9.7) | 9.9 | (9.4- 10.4) | 6.0 | (5.0- 6.9) | 4.9 | (3.5- 6.2) |
| Bushehr | 10.5 | (10.1- 11.0) | 11.6 | (11-12.2) | 8.4 | (8.0- 8.9) | 8.7 | (8.1- 9.3) | 4.4 | (3.1- 5.6) | 3.6 | (2.2- 5.0) |
| Zanjan | 11.2 | (10.9- 11.5) | 12.2 | (11.8-12.6) | 9.9 | (9.6- 10.2) | 10.4 | (10.0- 10.8) | 6.6 | (5.7- 7.5) | 6.8 | (5.8- 7.8) |
| Semnan | 9.7 | (9.5- 9.8) | 9.7 | (9.6-9.8) | 9.5 | (9.4- 9.7) | 9.6 | (9.5- 9.7) | 10.9 | (10.5- 11.3) | 10.5 | (10.3- 10.8) |
| Yazd | 9.3 | (9.1- 9.4) | 10.5 | (10.3-10.6) | 9.4 | (9.3- 9.6) | 10 | (9.8- 10.1) | 11.2 | (10.7- 11.7) | 10.9 | (10.5- 11.3) |
| Hormozgan | 9.0 | (8.8- 9.2) | 10.2 | (10-10.5) | 8.1 | (7.9- 8.3) | 9.2 | (9.0- 9.4) | 6.2 | (5.8- 6.7) | 7.2 | (6.7- 7.8) |
| Tehran | 9.2 | (9.2- 9.3) | 10.1 | (10-10.2) | 9.2 | (9.1- 9.2) | 9.7 | (9.6- 9.8) | 8.6 | (8.5- 8.8) | 8.6 | (8.3- 8.8) |
| Ardabil | 9.8 | (9.6- 9.9) | 9.6 | (9.4-9.8) | 9.2 | (9.1- 9.4) | 9.2 | (9.0- 9.4) | 7.5 | (7.0- 7.9) | 6.9 | (6.3- 7.5) |
| Qom | 7.3 | (7.2- 7.5) | 8.9 | (8.7-9.0) | 6.9 | (6.8- 7) | 8.2 | (8.1- 8.4) | 5.5 | (5.1- 5.8) | 6.9 | (6.5- 7.3) |
| Qazvin | 10.4 | (10.1- 10.7) | 11.2 | (11-11.4) | 9.2 | (8.9- 9.4) | 10.1 | (9.9- 10.3) | 6.6 | (5.8- 7.3) | 8.1 | (7.5- 8.6) |
| Golestan | 8.9 | (8.8- 9.1) | 9.8 | (9.8-9.9) | 9.7 | (9.6- 9.8) | 10.1 | (10.0- 10.1) | 10.1 | (9.8- 10.4) | 10.5 | (10.4- 10.7) |
| Khorasan_North | 8.6 | (8.4- 8.9) | 9.8 | (9.6-10.0) | 9.8 | (9.6- 10.1) | 10.7 | (10.6- 10.9) | 11.8 | (11.2- 12.4) | 12.2 | (11.8- 12.7) |
| Khorasan_South | 8.8 | (8.7- 8.9) | 10.3 | (10.1-10.5) | 9.1 | (9.0- 9.2) | 10.0 | (9.8- 10.2) | 8.4 | (8.1- 8.8) | 8.2 | (7.6- 9.0) |
| Alborz | 8.9 | (8.6- 9.1) | 10.0 | (9.9-10.2) | 9.5 | (9.3- 9.7) | 10.2 | (10.0- 10.3) | 11.7 | (11.1- 12.3) | 11.6 | (11.2- 12.0) |
| Iran | 9.6 | (9.4- 9.9) | 10.6 | (10.3-10.8) | 9.2 | (9- 9.4) | 9.8 | (9.6- 10.0) | 8.2 | (7.6- 8.8) | 8.3 | (7.7- 8.9) |

**Table 6. The age-standardized prevalence and projection of smoking in three time periods at sub-national level**

|  | 2001 | | | | 2016 | | | | 2030 | | | |
| --- | --- | --- | --- | --- | --- | --- | --- | --- | --- | --- | --- | --- |
|  | Female | | Male | | Female | | Male | | Female | | Male | |
| province | P | 95%CI | P | 95%CI | P | 95%CI | P | 95%CI | P | 95%CI | P | 95%CI |
| Markazi | 5.5 | (5.4- 5.6) | 39.8 | (37.8- 41.7) | 3.9 | (3.8- 4.0) | 29.8 | (27.8- 31.8) | 2.8 | (2.7- 2.9) | 22.6 | (20.5- 24.6) |
| Gilan | 3.3 | (3.2- 3.4) | 24.5 | (22.7- 26.4) | 2.3 | (2.2- 2.4) | 17.7 | (15.6- 19.6) | 1.6 | (1.5- 1.7) | 13.2 | (11.1- 15.2) |
| Mazandaran | 5.1 | (5.0- 5.2) | 37.0 | (35.1- 39.0) | 3.5 | (3.4- 3.6) | 26.1 | (24.2- 28.1) | 2.5 | (2.4- 2.6) | 18.8 | (16.7- 20.8) |
| Azerbaijan_East | 3.1 | (3.0- 3.2) | 22.7 | (20.8- 24.8) | 2.3 | (2.2- 2.4) | 16.6 | (14.7- 18.6) | 1.7 | (1.6- 1.8) | 12.2 | (10.1- 14.1) |
| Azerbaijan_West | 5.1 | (5.0- 5.2) | 38.4 | (36.4- 40.5) | 3.9 | (3.8- 4.0) | 28.3 | (26.4- 30.4) | 3.0 | (2.9- 3.1) | 21.1 | (19.1- 23.0) |
| Kermanshah | 4.9 | (4.8- 5.0) | 36.7 | (34.8- 38.6) | 3.8 | (3.7- 3.9) | 26.9 | (25.1- 28.8) | 2.9 | (2.8- 3.0) | 20.3 | (18.3- 22.1) |
| Khuzestan | 5.2 | (5.1- 5.3) | 39.3 | (37.4- 41.3) | 3.9 | (3.8- 4.0) | 28.5 | (26.7- 30.4) | 2.9 | (2.8- 3.0) | 20.4 | (18.6- 22.4) |
| Fars | 4.6 | (4.5- 4.7) | 34.1 | (32.3- 36.1) | 3.4 | (3.3- 3.5) | 24.6 | (22.7- 26.7) | 2.5 | (2.4- 2.6) | 17.9 | (15.9- 19.9) |
| Kerman | 3.5 | (3.4- 3.6) | 26.8 | (24.9- 28.8) | 2.6 | (2.5- 2.7) | 20.1 | (18.3- 22.1) | 1.9 | (1.8- 2.0) | 15.4 | (13.4- 17.2) |
| Khorasan_Razavi | 4.2 | (4.1- 4.3) | 31.6 | (29.6- 33.5) | 3.1 | (3.0- 3.2) | 23.0 | (21.0- 25.0) | 2.4 | (2.3- 2.5) | 17.1 | (15.0- 19.1) |
| Isfahan | 3.7 | (3.6- 3.8) | 27.6 | (25.6- 29.6) | 2.9 | (2.8- 3.0) | 21.6 | (19.7- 23.5) | 2.3 | (2.2- 2.4) | 17.3 | (15.2- 19.2) |
| Sistan&Baluchistan | 4.6 | (4.5- 4.7) | 34.8 | (32.7- 36.8) | 3.6 | (3.5- 3.7) | 26.0 | (24.1- 28.0) | 2.8 | (2.7- 2.9) | 19.8 | (17.9- 21.7) |
| Kurdistan | 4.3 | (4.2- 4.4) | 32.3 | (30.4- 34.4) | 3.3 | (3.2- 3.4) | 23.0 | (20.9- 24.9) | 2.6 | (2.5- 2.7) | 16.4 | (14.4- 18.2) |
| Hamadan | 4.4 | (4.3- 4.5) | 32.7 | (30.7- 34.7) | 3.2 | (3.1- 3.3) | 23.3 | (21.3- 25.5) | 2.3 | (2.2- 2.4) | 16.7 | (14.7- 18.7) |
| ChaharM&Bakhtiari | 4.0 | (3.9- 4.1) | 29.1 | (27.2- 31.0) | 2.9 | (2.8- 3.0) | 21.4 | (19.4- 23.2) | 2.1 | (2.0- 2.2) | 15.9 | (13.9- 17.9) |
| Lorestan | 4.0 | (3.9- 4.1) | 29.9 | (27.8- 31.9) | 2.9 | (2.8- 3.0) | 22.2 | (20.4- 24.2) | 2.2 | (2.1- 2.3) | 16.8 | (14.7- 18.8) |
| Ilam | 4.3 | (4.2- 4.4) | 31.3 | (29.4- 33.3) | 3.1 | (3.0- 3.2) | 22.2 | (20.2- 24.2) | 2.3 | (2.2- 2.4) | 15.9 | (14.0- 18.0) |
| Kohgiluyeh&BoyerA | 5.9 | (5.8- 6.0) | 42.7 | (40.6- 44.6) | 4.3 | (4.2- 4.4) | 29.8 | (27.9- 31.8) | 3.3 | (3.2- 3.4) | 21.5 | (19.6- 23.4) |
| Bushehr | 4.5 | (4.4- 4.6) | 32.7 | (30.9- 34.7) | 3.2 | (3.1- 3.3) | 23.3 | (21.2- 25.2) | 2.3 | (2.2- 2.4) | 16.8 | (14.8- 18.8) |
| Zanjan | 4.7 | (4.6- 4.8) | 35.2 | (33.3- 37.2) | 3.5 | (3.4- 3.6) | 25.7 | (23.9- 27.6) | 2.5 | (2.4- 2.6) | 18.6 | (16.7- 20.5) |
| Semnan | 3.5 | (3.4- 3.6) | 25.8 | (23.8- 27.8) | 2.4 | (2.3- 2.5) | 18.8 | (16.8- 20.8) | 1.7 | (1.6- 1.8) | 14.2 | (12.2- 15.9) |
| Yazd | 4.1 | (4.0- 4.2) | 30.0 | (28.0- 31.9) | 2.9 | (2.8- 3.0) | 20.9 | (18.9- 23.0) | 2.0 | (1.9- 2.1) | 14.5 | (12.4- 16.4) |
| Hormozgan | 4.2 | (4.1- 4.3) | 31.8 | (29.9- 33.6) | 3.0 | (2.9- 3.1) | 22.8 | (20.8- 24.6) | 2.2 | (2.1- 2.3) | 16.8 | (14.8- 18.8) |
| Tehran | 3.3 | (3.2- 3.4) | 24.9 | (23.0- 26.9) | 2.5 | (2.4- 2.6) | 19.9 | (18.0- 21.9) | 2.0 | (1.9- 2.1) | 16.5 | (14.4- 18.5) |
| Ardabil | 6.0 | (5.9- 6.1) | 43.7 | (41.7- 45.6) | 4.4 | (4.2- 4.4) | 30.7 | (28.7- 32.7) | 3.2 | (3.1- 3.3) | 21.9 | (19.9- 23.8) |
| Qom | 4.3 | (4.2- 4.4) | 31.1 | (29.1- 32.9) | 3.2 | (3.1- 3.3) | 23.6 | (21.6- 25.5) | 2.3 | (2.2- 2.4) | 17.5 | (15.5- 19.4) |
| Qazvin | 5.2 | (5.1- 5.3) | 39.0 | (37.0- 41.0) | 3.9 | (3.8- 4.0) | 30.2 | (28.2- 32.2) | 3.0 | (2.9- 3.1) | 24.3 | (22.4- 26.2) |
| Golestan | 4.9 | (4.8- 5.0) | 35.5 | (33.6- 37.4) | 3.5 | (3.4- 3.6) | 24.7 | (22.6- 26.8) | 2.6 | (2.5- 2.7) | 17.5 | (15.5- 19.4) |
| Khorasan_North | 4.0 | (3.9- 4.1) | 30.6 | (28.4- 32.5) | 2.9 | (2.8- 3.0) | 20.8 | (19.0- 22.7) | 2.1 | (2.0- 2.2) | 14.4 | (12.5- 16.4) |
| Khorasan_South | 4.4 | (4.3- 4.5) | 32.9 | (31.1- 34.9) | 3.1 | (3.0- 3.2) | 21.9 | (20.0- 23.9) | 2.2 | (2.1- 2.3) | 15.1 | (13.1- 16.9) |
| Alborz | 4.2 | (4.1- 4.3) | 31.7 | (29.6- 33.6) | 3.1 | (3.0- 3.2) | 24.4 | (22.5- 26.3) | 2.3 | (2.2- 2.4) | 19.2 | (17.2- 21.1) |
| Iran | 4.4 | (4.3- 4.5) | 32.8 | (30.9- 34.8) | 3.2 | (3.1- 3.3) | 23.8 | (21.8- 25.8) | 2.4 | (2.3- 2.5) | 17.6 | (15.6- 19.5) |

**Appendix 4: Avoidable deaths at sub-national level**

| **Table 7. The number of avoidable deaths based on theoretical minimum risk exposure level scenario at sub-national level** | | | | | | | | | | | | | | | | | | | | | | | | | | | | | | | | |
| --- | --- | --- | --- | --- | --- | --- | --- | --- | --- | --- | --- | --- | --- | --- | --- | --- | --- | --- | --- | --- | --- | --- | --- | --- | --- | --- | --- | --- | --- | --- | --- | --- |
|  | FPG | | | | HTN | | | | Overweight and Obesity | | | | | | Physical inactivity | | | | | | Salt | | | | Smoking | | | | | | | |
|  | CVDs | | Diabetes | | CVDs | | Diabetes | | Cancers | | CVDs | | Diabetes | | Cancers | | CVDs | | Diabetes | | Cancer | | Indirect on CVDs | | Cancers | | CVDs | | Diabetes | | Asthma and cOPD | |
| Province | F | M | F | M | F | M | F | M | F | M | F | M | F | M | F | M | F | M | F | M | F | M | F | M | F | M | F | M | F | M | F | M |
| Markazi | 210 | 363 | 62 | 56 | 590 | 519 | 2 | 1 | 43 | 23 | 155 | 183 | 49 | 36 | 26 | 19 | 24 | 8 | 88 | 130 | 8 | 13 | 225 | 341 | 39 | 18 | 154 | 95 | 5 | 1 | 15 | 9 |
| Gilan | 446 | 737 | 96 | 82 | 1271 | 979 | 5 | 4 | 67 | 33 | 303 | 352 | 77 | 54 | 41 | 29 | 45 | 11 | 184 | 264 | 15 | 24 | 504 | 707 | 59 | 28 | 275 | 174 | 6 | 2 | 24 | 14 |
| Mazandaran | 479 | 870 | 73 | 66 | 1113 | 921 | 6 | 6 | 66 | 36 | 314 | 408 | 59 | 45 | 35 | 27 | 33 | 9 | 216 | 354 | 10 | 10 | 525 | 783 | 48 | 22 | 279 | 167 | 4 | 1 | 22 | 13 |
| Azerbaijan_East | 514 | 956 | 96 | 93 | 1435 | 1352 | 6 | 5 | 86 | 40 | 357 | 463 | 76 | 61 | 48 | 41 | 41 | 15 | 249 | 439 | 13 | 21 | 573 | 930 | 74 | 32 | 362 | 211 | 7 | 2 | 31 | 17 |
| Azerbaijan_West | 340 | 640 | 53 | 52 | 937 | 847 | 4 | 4 | 60 | 25 | 240 | 316 | 42 | 34 | 21 | 16 | 15 | 5 | 133 | 206 | 7 | 13 | 384 | 598 | 45 | 20 | 240 | 141 | 4 | 1 | 20 | 12 |
| Kermanshah | 248 | 450 | 59 | 58 | 699 | 636 | 2 | 2 | 53 | 24 | 184 | 227 | 47 | 38 | 26 | 20 | 19 | 8 | 102 | 162 | 11 | 20 | 301 | 459 | 41 | 19 | 184 | 112 | 5 | 1 | 17 | 10 |
| Khuzestan | 549 | 1054 | 132 | 133 | 1568 | 1527 | 5 | 5 | 111 | 55 | 394 | 519 | 105 | 89 | 59 | 50 | 48 | 20 | 229 | 403 | 19 | 35 | 641 | 1047 | 94 | 41 | 412 | 239 | 11 | 3 | 38 | 22 |
| Fars | 642 | 1225 | 174 | 173 | 1455 | 1504 | 5 | 5 | 121 | 58 | 448 | 547 | 133 | 105 | 59 | 39 | 50 | 16 | 212 | 289 | 24 | 42 | 638 | 1116 | 111 | 49 | 485 | 280 | 13 | 4 | 45 | 26 |
| Kerman | 294 | 506 | 57 | 51 | 641 | 547 | 4 | 3 | 44 | 18 | 190 | 209 | 41 | 27 | 23 | 16 | 19 | 5 | 129 | 187 | 4 | 4 | 327 | 492 | 37 | 16 | 204 | 121 | 3 | 1 | 19 | 10 |
| Khorasan_Razavi | 730 | 1310 | 179 | 172 | 2045 | 1993 | 7 | 7 | 140 | 66 | 541 | 662 | 140 | 109 | 70 | 50 | 59 | 19 | 292 | 426 | 26 | 53 | 826 | 1396 | 123 | 56 | 557 | 334 | 13 | 4 | 51 | 30 |
| Isfahan | 761 | 1367 | 188 | 176 | 2027 | 1898 | 5 | 5 | 155 | 81 | 554 | 687 | 149 | 117 | 91 | 74 | 78 | 30 | 342 | 576 | 24 | 46 | 705 | 1171 | 135 | 62 | 562 | 340 | 15 | 4 | 53 | 30 |
| Sistan&Baluchistan | 171 | 313 | 44 | 41 | 359 | 329 | 2 | 1 | 37 | 14 | 116 | 122 | 31 | 21 | 19 | 14 | 11 | 5 | 75 | 117 | 6 | 12 | 193 | 298 | 27 | 13 | 133 | 82 | 3 | 1 | 14 | 9 |
| Kurdistan | 167 | 333 | 23 | 26 | 510 | 531 | 2 | 2 | 28 | 11 | 118 | 164 | 18 | 16 | 10 | 8 | 7 | 3 | 67 | 115 | 6 | 11 | 196 | 335 | 23 | 10 | 127 | 70 | 2 | 0 | 10 | 6 |
| Hamadan | 212 | 381 | 53 | 48 | 578 | 458 | 2 | 2 | 42 | 19 | 158 | 190 | 42 | 31 | 26 | 20 | 21 | 7 | 105 | 176 | 7 | 10 | 263 | 367 | 32 | 15 | 153 | 93 | 4 | 1 | 14 | 8 |
| ChaharM&Bakhtiari | 102 | 187 | 27 | 26 | 267 | 243 | 1 | 1 | 21 | 11 | 77 | 96 | 21 | 16 | 13 | 11 | 11 | 4 | 52 | 89 | 4 | 6 | 139 | 208 | 17 | 7 | 78 | 45 | 2 | 1 | 8 | 4 |
| Lorestan | 189 | 311 | 30 | 26 | 550 | 419 | 3 | 2 | 29 | 12 | 132 | 153 | 24 | 17 | 15 | 11 | 12 | 4 | 95 | 150 | 5 | 10 | 229 | 315 | 22 | 10 | 121 | 77 | 2 | 1 | 10 | 6 |
| Ilam | 65 | 123 | 17 | 17 | 186 | 167 | 1 | 1 | 13 | 6 | 49 | 62 | 13 | 10 | 8 | 6 | 6 | 2 | 31 | 53 | 2 | 4 | 84 | 125 | 11 | 5 | 52 | 30 | 1 | 0 | 5 | 3 |
| Kohgiluyeh&BoyerA | 66 | 132 | 13 | 13 | 175 | 170 | 1 | 1 | 13 | 6 | 49 | 68 | 10 | 9 | 6 | 5 | 4 | 2 | 32 | 59 | 2 | 2 | 80 | 129 | 9 | 4 | 50 | 28 | 1 | 0 | 4 | 3 |
| Bushehr | 128 | 304 | 55 | 71 | 346 | 485 | 1 | 1 | 34 | 27 | 109 | 174 | 43 | 48 | 27 | 31 | 23 | 14 | 66 | 142 | 2 | 2 | 177 | 328 | 40 | 13 | 139 | 64 | 6 | 1 | 15 | 7 |
| Zanjan | 106 | 191 | 7 | 7 | 346 | 315 | 2 | 2 | 10 | 4 | 70 | 93 | 6 | 5 | 3 | 3 | 3 | 1 | 52 | 86 | 2 | 5 | 114 | 177 | 9 | 4 | 63 | 36 | 0 | 0 | 4 | 2 |
| Semnan | 107 | 179 | 26 | 23 | 286 | 242 | 1 | 1 | 18 | 10 | 76 | 93 | 21 | 15 | 12 | 8 | 13 | 3 | 47 | 69 | 4 | 7 | 110 | 173 | 15 | 7 | 69 | 42 | 2 | 1 | 6 | 3 |
| Yazd | 156 | 316 | 32 | 34 | 404 | 423 | 1 | 1 | 25 | 14 | 109 | 159 | 26 | 23 | 14 | 11 | 15 | 5 | 62 | 108 | 6 | 12 | 139 | 265 | 24 | 10 | 113 | 62 | 3 | 1 | 9 | 5 |
| Hormozgan | 167 | 303 | 79 | 74 | 438 | 422 | 1 | 1 | 48 | 27 | 143 | 165 | 60 | 45 | 32 | 23 | 23 | 9 | 71 | 106 | 5 | 11 | 258 | 409 | 38 | 18 | 149 | 88 | 6 | 2 | 18 | 10 |
| Tehran | 2256 | 3656 | 560 | 469 | 5907 | 5556 | 15 | 15 | 418 | 232 | 1712 | 2069 | 465 | 336 | 257 | 182 | 281 | 75 | 1011 | 1468 | 75 | 130 | 1873 | 3408 | 357 | 179 | 1515 | 976 | 39 | 13 | 137 | 78 |
| Ardabil | 145 | 285 | 27 | 28 | 429 | 442 | 2 | 2 | 27 | 14 | 112 | 165 | 22 | 20 | 11 | 9 | 9 | 3 | 61 | 101 | 4 | 8 | 182 | 306 | 22 | 9 | 109 | 61 | 2 | 1 | 9 | 5 |
| Qom | 180 | 323 | 37 | 35 | 559 | 539 | 1 | 2 | 34 | 18 | 134 | 183 | 31 | 26 | 17 | 13 | 17 | 5 | 75 | 122 | 3 | 9 | 172 | 308 | 29 | 13 | 127 | 76 | 3 | 1 | 11 | 6 |
| Qazvin | 165 | 287 | 45 | 41 | 426 | 391 | 1 | 1 | 39 | 23 | 131 | 164 | 36 | 29 | 19 | 15 | 15 | 6 | 69 | 105 | 5 | 11 | 174 | 273 | 30 | 13 | 125 | 75 | 3 | 1 | 13 | 7 |
| Golestan | 212 | 397 | 62 | 59 | 493 | 457 | 2 | 2 | 52 | 28 | 176 | 232 | 51 | 43 | 26 | 20 | 20 | 7 | 89 | 144 | 9 | 17 | 279 | 430 | 34 | 16 | 155 | 94 | 4 | 1 | 15 | 9 |
| Khorasan_North | 83 | 156 | 15 | 15 | 224 | 215 | 1 | 1 | 15 | 7 | 60 | 82 | 12 | 10 | 8 | 7 | 6 | 2 | 47 | 89 | 4 | 7 | 110 | 176 | 11 | 4 | 58 | 34 | 1 | 0 | 5 | 3 |
| Khorasan_South | 77 | 143 | 19 | 18 | 182 | 170 | 1 | 1 | 15 | 7 | 56 | 71 | 15 | 12 | 9 | 8 | 8 | 3 | 40 | 69 | 3 | 5 | 90 | 139 | 11 | 5 | 56 | 33 | 1 | 0 | 5 | 3 |
| Alborz | 476 | 813 | 118 | 107 | 1351 | 1332 | 4 | 3 | 85 | 55 | 355 | 472 | 99 | 79 | 58 | 46 | 67 | 20 | 222 | 347 | 18 | 35 | 436 | 752 | 81 | 37 | 330 | 199 | 9 | 3 | 29 | 16 |

| **Table 8. The number of avoidable deaths based on 2010 baseline at sub-national level** | | | | | | | | | | | | | | | | | | | | | | | | | | | | | | | | |
| --- | --- | --- | --- | --- | --- | --- | --- | --- | --- | --- | --- | --- | --- | --- | --- | --- | --- | --- | --- | --- | --- | --- | --- | --- | --- | --- | --- | --- | --- | --- | --- | --- |
|  | FPG | | | | HTN | | | |  | | | | | | Physical inactivity | | | | | | Salt | | | | Smoking | | | | | | | |
|  | CVDs | | Diabetes | | CVDs | | Diabetes | | Cancers | | CVDs | | Diabetes | | Cancers | | CVDs | | Diabetes | | Cancer | | Indirect on CVDs | | Cancers | | CVDs | | Diabetes | | Asthma and cOPD | |
| Province | F | M | F | M | F | M | F | M | F | M | F | M | F | M | F | M | F | M | F | M | F | M | F | M | F | M | F | M | F | M | F | M |
| Markazi | 11 | 28 | 34 | 27 | 189 | 195 | 1 | 1 | 21 | 15 | 48 | 72 | 14 | 15 | 21 | 10 | 73 | 161 | 22 | 23 | 2 | 0 | 141 | 171 | 5 | 9 | 24 | 40 | 0 | 1 | 2 | 4 |
| Gilan | 15 | 33 | 46 | 33 | 344 | 381 | 2 | 1 | 31 | 22 | 98 | 152 | 21 | 22 | 37 | 13 | 154 | 310 | 34 | 34 | 5 | 4 | 274 | 360 | 6 | 13 | 40 | 63 | 1 | 2 | 3 | 5 |
| Mazandaran | 5 | 11 | 27 | 21 | 435 | 441 | 1 | 1 | 26 | 20 | 89 | 166 | 13 | 17 | 24 | 9 | 154 | 336 | 25 | 26 | 3 | 16 | 285 | 380 | 3 | 7 | 26 | 43 | 0 | 1 | 2 | 3 |
| Azerbaijan_East | 21 | 44 | 30 | 26 | 477 | 505 | 1 | 1 | 38 | 25 | 110 | 189 | 20 | 23 | 26 | 12 | 153 | 350 | 30 | 33 | 1 | 9 | 307 | 442 | 6 | 13 | 39 | 68 | 0 | 1 | 3 | 6 |
| Azerbaijan_West | 22 | 54 | 8 | 6 | 424 | 369 | 1 | 1 | 23 | 15 | 73 | 134 | 11 | 14 | 15 | 8 | 127 | 301 | 21 | 23 | 4 | 9 | 220 | 285 | 4 | 7 | 26 | 41 | 0 | 1 | 2 | 4 |
| Kermanshah | 11 | 33 | 21 | 19 | 249 | 243 | 1 | 1 | 21 | 14 | 53 | 87 | 11 | 14 | 17 | 9 | 87 | 201 | 22 | 24 | 4 | 6 | 156 | 211 | 4 | 8 | 24 | 38 | 0 | 1 | 2 | 4 |
| Khuzestan | 7 | 27 | 59 | 60 | 526 | 544 | 2 | 2 | 45 | 30 | 113 | 205 | 27 | 35 | 38 | 22 | 180 | 435 | 47 | 54 | 5 | 5 | 340 | 471 | 9 | 19 | 54 | 90 | 1 | 3 | 5 | 8 |
| Fars | 20 | 65 | 85 | 83 | 517 | 584 | 2 | 2 | 60 | 30 | 139 | 179 | 40 | 37 | 63 | 35 | 269 | 661 | 74 | 89 | 6 | 4 | 370 | 548 | 11 | 23 | 61 | 105 | 1 | 3 | 5 | 10 |
| Kerman | 16 | 26 | 14 | 8 | 319 | 341 | 1 | 0 | 19 | 8 | 66 | 66 | 13 | 10 | 17 | 7 | 119 | 256 | 22 | 22 | 5 | 15 | 188 | 235 | 2 | 5 | 16 | 27 | 0 | 1 | 1 | 2 |
| Khorasan_Razavi | 76 | 160 | 73 | 64 | 723 | 725 | 3 | 2 | 70 | 36 | 177 | 249 | 41 | 42 | 60 | 30 | 294 | 671 | 71 | 78 | 3 | 14 | 499 | 678 | 11 | 23 | 68 | 112 | 1 | 3 | 6 | 10 |
| Isfahan | 20 | 45 | 87 | 75 | 653 | 724 | 3 | 2 | 57 | 40 | 144 | 216 | 34 | 38 | 52 | 26 | 224 | 496 | 60 | 64 | 2 | 3 | 440 | 606 | 15 | 29 | 80 | 130 | 1 | 4 | 7 | 12 |
| Sistan&Baluchistan | 10 | 26 | 9 | 6 | 247 | 215 | 1 | 0 | 17 | 5 | 41 | 36 | 11 | 8 | 10 | 6 | 62 | 142 | 16 | 17 | 1 | 0 | 145 | 177 | 2 | 5 | 17 | 25 | 0 | 1 | 2 | 3 |
| Kurdistan | 19 | 45 | 1 | 2 | 260 | 230 | 0 | 0 | 11 | 5 | 40 | 71 | 5 | 7 | 6 | 4 | 62 | 159 | 9 | 11 | 0 | 1 | 115 | 166 | 2 | 4 | 13 | 23 | 0 | 0 | 1 | 2 |
| Hamadan | 14 | 34 | 24 | 19 | 208 | 190 | 1 | 0 | 16 | 14 | 50 | 69 | 11 | 12 | 15 | 7 | 70 | 155 | 17 | 18 | 0 | 3 | 168 | 206 | 3 | 6 | 19 | 30 | 0 | 1 | 2 | 3 |
| ChaharM&Bakhtiari | 6 | 15 | 11 | 9 | 96 | 94 | 0 | 0 | 7 | 2 | 22 | 34 | 5 | 5 | 7 | 3 | 33 | 74 | 8 | 9 | 0 | 0 | 73 | 90 | 1 | 3 | 8 | 16 | 0 | 0 | 1 | 2 |
| Lorestan | 17 | 32 | 4 | 1 | 241 | 210 | 0 | 0 | 12 | 5 | 42 | 58 | 6 | 6 | 7 | 3 | 61 | 125 | 9 | 9 | 0 | 1 | 138 | 159 | 2 | 3 | 12 | 19 | 0 | 0 | 1 | 2 |
| Ilam | 8 | 18 | 7 | 6 | 80 | 72 | 0 | 0 | 5 | 1 | 16 | 21 | 4 | 4 | 5 | 3 | 24 | 55 | 6 | 7 | 0 | 0 | 58 | 69 | 1 | 2 | 6 | 11 | 0 | 0 | 1 | 1 |
| Kohgiluyeh&BoyerA | 9 | 22 | 3 | 3 | 78 | 70 | 0 | 0 | 5 | 2 | 16 | 28 | 2 | 3 | 3 | 2 | 22 | 55 | 4 | 5 | 1 | 2 | 48 | 69 | 1 | 2 | 6 | 10 | 0 | 0 | 1 | 1 |
| Bushehr | 8 | 38 | 32 | 43 | 102 | 137 | 1 | 1 | 13 | 15 | 31 | 59 | 11 | 17 | 14 | 12 | 41 | 115 | 17 | 25 | 2 | 8 | 78 | 121 | 3 | 11 | 15 | 39 | 0 | 2 | 2 | 4 |
| Zanjan | 12 | 23 | -2 | -3 | 205 | 182 | 0 | 0 | 3 | 0 | 24 | 41 | 2 | 2 | 3 | 1 | 37 | 81 | 2 | 3 | 1 | 1 | 60 | 75 | 0 | 1 | 3 | 6 | 0 | 0 | 0 | 0 |
| Semnan | 1 | 4 | 13 | 10 | 78 | 83 | 0 | 0 | 5 | 3 | 20 | 30 | 5 | 5 | 11 | 3 | 36 | 72 | 9 | 9 | 2 | 3 | 69 | 87 | 1 | 3 | 9 | 14 | 0 | 0 | 1 | 1 |
| Yazd | 1 | 4 | 14 | 14 | 132 | 167 | 1 | 1 | 10 | 7 | 30 | 54 | 5 | 7 | 14 | 6 | 55 | 137 | 12 | 14 | 2 | 4 | 78 | 127 | 2 | 4 | 12 | 20 | 0 | 0 | 1 | 2 |
| Hormozgan | 18 | 38 | 48 | 42 | 165 | 184 | 1 | 1 | 22 | 9 | 40 | 41 | 17 | 14 | 22 | 13 | 67 | 152 | 30 | 33 | 0 | 1 | 125 | 170 | 5 | 9 | 24 | 39 | 1 | 2 | 3 | 4 |
| Tehran | 70 | 198 | 255 | 187 | 1569 | 1798 | 8 | 5 | 129 | 89 | 352 | 494 | 72 | 76 | 208 | 75 | 749 | 1468 | 190 | 183 | 19 | 19 | 1157 | 1641 | 37 | 67 | 199 | 307 | 3 | 8 | 15 | 26 |
| Ardabil | 13 | 33 | 7 | 7 | 162 | 172 | 0 | 0 | 8 | 8 | 29 | 60 | 4 | 7 | 9 | 4 | 54 | 133 | 10 | 12 | 0 | 0 | 86 | 130 | 2 | 4 | 11 | 22 | 0 | 0 | 1 | 2 |
| Qom | 3 | 8 | 15 | 13 | 157 | 172 | 1 | 0 | 11 | 10 | 31 | 58 | 5 | 7 | 13 | 6 | 58 | 131 | 13 | 14 | 0 | 1 | 106 | 150 | 3 | 5 | 15 | 25 | 0 | 1 | 1 | 2 |
| Qazvin | 6 | 13 | 21 | 17 | 151 | 158 | 1 | 0 | 12 | 10 | 32 | 50 | 7 | 8 | 13 | 6 | 56 | 120 | 16 | 17 | 1 | 1 | 110 | 140 | 3 | 7 | 18 | 31 | 0 | 1 | 2 | 3 |
| Golestan | 9 | 24 | 31 | 29 | 213 | 232 | 1 | 1 | 18 | 15 | 45 | 74 | 11 | 12 | 17 | 10 | 79 | 186 | 23 | 26 | 3 | 5 | 155 | 224 | 3 | 7 | 19 | 31 | 0 | 1 | 2 | 3 |
| Khorasan_North | 9 | 18 | 4 | 4 | 102 | 86 | 0 | 0 | 5 | 2 | 19 | 33 | 3 | 4 | 3 | 2 | 23 | 53 | 4 | 4 | 2 | 3 | 63 | 86 | 1 | 2 | 6 | 10 | 0 | 0 | 0 | 1 |
| Khorasan_South | 8 | 15 | 7 | 7 | 82 | 70 | 0 | 0 | 5 | 1 | 20 | 24 | 4 | 4 | 5 | 2 | 25 | 58 | 6 | 7 | 1 | 0 | 66 | 78 | 1 | 2 | 6 | 11 | 0 | 0 | 1 | 1 |
| Alborz | 8 | 25 | 56 | 46 | 158 | 172 | 1 | 1 | 27 | 25 | 75 | 135 | 16 | 20 | 43 | 16 | 143 | 294 | 37 | 38 | 8 | 13 | 149 | 214 | 8 | 18 | 42 | 74 | 1 | 3 | 3 | 6 |

| **Table 9. The number of avoidable deaths based on 2015 scenario at sub-national level** | | | | | | | | | | | | | | | | | | | | | | | | | | | | | | | | |
| --- | --- | --- | --- | --- | --- | --- | --- | --- | --- | --- | --- | --- | --- | --- | --- | --- | --- | --- | --- | --- | --- | --- | --- | --- | --- | --- | --- | --- | --- | --- | --- | --- |
|  | FPG | | | | HTN | | | | Overweight and Obesity | | | | | | Physical inactivity | | | | | | Salt | | | | Smoking | | | | | | | |
|  | CVDs | | Diabetes | | CVDs | | Diabetes | | Cancers | | CVDs | | Diabetes | | Cancers | | CVDs | | Diabetes | | Cancer | | Indirect on CVDs | | Cancers | | CVDs | | Diabetes | | Asthma and cOPD | |
| Province | F | M | F | M | F | M | F | M | F | M | F | M | F | M | F | M | F | M | F | M | F | M | F | M | F | M | F | M | F | M | F | M |
| Markazi | 3 | 12 | 25 | 20 | 177 | 187 | 1 | 1 | 7 | 7 | 39 | 67 | 5 | 6 | 15 | 7 | 56 | 118 | 17 | 18 | 2 | 4 | 107 | 117 | 5 | 10 | 26 | 41 | 0 | 1 | 2 | 4 |
| Gilan | 4 | 11 | 33 | 23 | 309 | 356 | 1 | 1 | 12 | 9 | 77 | 131 | 8 | 10 | 29 | 10 | 115 | 236 | 26 | 26 | 4 | 8 | 176 | 218 | 7 | 14 | 44 | 68 | 1 | 2 | 4 | 6 |
| Mazandaran | 0 | 3 | 18 | 13 | 380 | 400 | 1 | 1 | 10 | 8 | 67 | 138 | 5 | 7 | 19 | 7 | 117 | 259 | 19 | 20 | 5 | 9 | 179 | 214 | 4 | 9 | 32 | 53 | 0 | 1 | 2 | 4 |
| Azerbaijan_East | 7 | 17 | 17 | 16 | 456 | 474 | 1 | 1 | 13 | 8 | 80 | 156 | 6 | 10 | 19 | 9 | 117 | 268 | 22 | 25 | 6 | 11 | 212 | 262 | 7 | 15 | 44 | 76 | 1 | 1 | 4 | 6 |
| Azerbaijan_West | 8 | 24 | 2 | 2 | 423 | 383 | 1 | 1 | 7 | 7 | 51 | 108 | 3 | 5 | 12 | 6 | 97 | 229 | 16 | 18 | 4 | 8 | 155 | 174 | 4 | 9 | 29 | 47 | 0 | 1 | 2 | 4 |
| Kermanshah | 5 | 9 | 13 | 12 | 215 | 210 | 1 | 1 | 8 | 7 | 39 | 74 | 4 | 6 | 13 | 7 | 66 | 152 | 17 | 19 | 3 | 5 | 106 | 124 | 4 | 9 | 26 | 42 | 0 | 1 | 2 | 4 |
| Khuzestan | 0 | 6 | 40 | 42 | 419 | 422 | 2 | 1 | 20 | 12 | 90 | 182 | 11 | 15 | 29 | 16 | 138 | 329 | 35 | 41 | 6 | 12 | 228 | 268 | 10 | 21 | 59 | 99 | 1 | 3 | 5 | 9 |
| Fars | 5 | 14 | 59 | 60 | 502 | 543 | 2 | 2 | 21 | 14 | 118 | 184 | 15 | 17 | 48 | 27 | 205 | 496 | 56 | 67 | 8 | 15 | 287 | 346 | 12 | 25 | 66 | 115 | 1 | 3 | 6 | 11 |
| Kerman | 3 | 5 | 6 | 2 | 291 | 315 | 1 | 0 | 7 | 4 | 51 | 65 | 5 | 4 | 13 | 5 | 91 | 194 | 16 | 17 | 3 | 4 | 131 | 140 | 3 | 5 | 20 | 32 | 0 | 1 | 2 | 3 |
| Khorasan_Razavi | 28 | 61 | 47 | 42 | 695 | 670 | 3 | 2 | 21 | 17 | 136 | 227 | 15 | 18 | 45 | 23 | 224 | 509 | 54 | 60 | 9 | 16 | 373 | 406 | 12 | 26 | 74 | 124 | 1 | 4 | 6 | 11 |
| Isfahan | 5 | 27 | 60 | 53 | 670 | 731 | 3 | 2 | 22 | 19 | 120 | 205 | 13 | 16 | 40 | 20 | 171 | 379 | 45 | 49 | 9 | 16 | 360 | 415 | 15 | 31 | 84 | 134 | 1 | 4 | 7 | 12 |
| Sistan&Baluchistan | 3 | 10 | 2 | 2 | 268 | 242 | 1 | 0 | 7 | 1 | 33 | 38 | 4 | 3 | 7 | 4 | 48 | 107 | 12 | 13 | 2 | 4 | 122 | 125 | 3 | 5 | 18 | 28 | 0 | 1 | 2 | 3 |
| Kurdistan | 7 | 21 | 1 | 0 | 228 | 198 | 0 | 0 | 4 | 1 | 27 | 56 | 2 | 3 | 5 | 3 | 48 | 120 | 7 | 8 | 2 | 4 | 77 | 95 | 2 | 5 | 15 | 27 | 0 | 0 | 1 | 2 |
| Hamadan | 6 | 13 | 16 | 13 | 223 | 229 | 1 | 1 | 7 | 6 | 39 | 62 | 4 | 5 | 11 | 5 | 53 | 118 | 13 | 14 | 3 | 5 | 122 | 139 | 4 | 7 | 22 | 34 | 0 | 1 | 2 | 3 |
| ChaharM&Bakhtiari | 2 | 6 | 7 | 6 | 85 | 87 | 0 | 0 | 3 | 1 | 19 | 31 | 2 | 2 | 5 | 3 | 25 | 58 | 6 | 7 | 1 | 3 | 46 | 48 | 2 | 4 | 10 | 17 | 0 | 0 | 1 | 2 |
| Lorestan | 7 | 13 | 1 | 1 | 224 | 210 | 0 | 0 | 3 | 1 | 31 | 49 | 2 | 2 | 6 | 2 | 46 | 94 | 7 | 7 | 2 | 4 | 92 | 95 | 2 | 4 | 14 | 22 | 0 | 0 | 1 | 2 |
| Ilam | 2 | 7 | 4 | 4 | 73 | 70 | 0 | 0 | 1 | 1 | 13 | 21 | 1 | 2 | 3 | 2 | 18 | 42 | 4 | 5 | 1 | 2 | 41 | 44 | 1 | 3 | 7 | 12 | 0 | 0 | 1 | 1 |
| Kohgiluyeh&BoyerA | 4 | 11 | 1 | 1 | 87 | 80 | 0 | 0 | 1 | 1 | 13 | 23 | 1 | 1 | 2 | 1 | 17 | 42 | 3 | 4 | 1 | 2 | 36 | 43 | 1 | 2 | 7 | 12 | 0 | 0 | 1 | 1 |
| Bushehr | 2 | 13 | 23 | 34 | 73 | 97 | 1 | 1 | 5 | 7 | 25 | 59 | 4 | 7 | 11 | 9 | 32 | 87 | 13 | 19 | 2 | 2 | 50 | 68 | 3 | 12 | 16 | 41 | 0 | 2 | 2 | 4 |
| Zanjan | 6 | 9 | 3 | 3 | 165 | 142 | 0 | 0 | 1 | 0 | 16 | 30 | 0 | 1 | 1 | 1 | 28 | 61 | 2 | 2 | 1 | 2 | 36 | 40 | 0 | 1 | 4 | 9 | 0 | 0 | 0 | 1 |
| Semnan | 0 | 1 | 10 | 7 | 79 | 83 | 0 | 0 | 1 | 1 | 17 | 28 | 2 | 2 | 8 | 3 | 28 | 55 | 7 | 7 | 1 | 2 | 49 | 54 | 2 | 3 | 10 | 16 | 0 | 0 | 1 | 1 |
| Yazd | 0 | 1 | 9 | 9 | 100 | 119 | 0 | 0 | 4 | 3 | 22 | 48 | 2 | 3 | 10 | 4 | 42 | 104 | 9 | 11 | 1 | 3 | 57 | 76 | 2 | 5 | 14 | 23 | 0 | 1 | 1 | 2 |
| Hormozgan | 6 | 16 | 37 | 32 | 112 | 115 | 1 | 1 | 10 | 7 | 34 | 50 | 7 | 7 | 17 | 10 | 51 | 115 | 23 | 25 | 2 | 4 | 82 | 91 | 5 | 10 | 25 | 41 | 1 | 2 | 3 | 5 |
| Tehran | 16 | 110 | 174 | 128 | 1707 | 1798 | 8 | 5 | 48 | 43 | 293 | 475 | 29 | 35 | 159 | 57 | 571 | 1118 | 145 | 139 | 25 | 44 | 966 | 1053 | 40 | 69 | 214 | 314 | 3 | 8 | 17 | 27 |
| Ardabil | 4 | 13 | 4 | 4 | 110 | 112 | 0 | 0 | 3 | 1 | 20 | 50 | 1 | 2 | 6 | 3 | 42 | 101 | 8 | 9 | 2 | 4 | 48 | 65 | 2 | 5 | 13 | 25 | 0 | 1 | 1 | 2 |
| Qom | 0 | 0 | 10 | 8 | 149 | 155 | 1 | 0 | 5 | 5 | 25 | 49 | 2 | 3 | 10 | 4 | 43 | 100 | 10 | 11 | 2 | 4 | 81 | 92 | 3 | 6 | 17 | 28 | 0 | 1 | 1 | 2 |
| Qazvin | 2 | 4 | 14 | 11 | 157 | 168 | 1 | 0 | 5 | 6 | 26 | 47 | 3 | 3 | 9 | 5 | 43 | 91 | 12 | 13 | 2 | 4 | 87 | 94 | 3 | 7 | 19 | 31 | 0 | 1 | 2 | 3 |
| Golestan | 3 | 7 | 22 | 21 | 171 | 180 | 1 | 1 | 7 | 7 | 35 | 66 | 4 | 5 | 14 | 7 | 60 | 140 | 18 | 19 | 2 | 5 | 101 | 129 | 4 | 7 | 22 | 35 | 0 | 1 | 2 | 3 |
| Khorasan_North | 3 | 9 | 2 | 2 | 99 | 84 | 0 | 0 | 2 | 1 | 15 | 27 | 1 | 1 | 3 | 1 | 17 | 38 | 3 | 3 | 1 | 2 | 40 | 47 | 1 | 2 | 7 | 12 | 0 | 0 | 1 | 1 |
| Khorasan_South | 2 | 5 | 4 | 4 | 88 | 80 | 0 | 0 | 2 | 1 | 16 | 22 | 2 | 2 | 4 | 2 | 19 | 44 | 5 | 5 | 1 | 2 | 51 | 53 | 1 | 2 | 7 | 12 | 0 | 0 | 1 | 1 |
| Alborz | 2 | 9 | 38 | 33 | 233 | 246 | 1 | 1 | 10 | 11 | 62 | 120 | 5 | 9 | 33 | 13 | 109 | 223 | 28 | 29 | 4 | 9 | 114 | 112 | 9 | 18 | 46 | 79 | 1 | 3 | 4 | 7 |

| **Table 10. The prevalence of alcohol consumption in the latest month (Steps survey in 2016)** | | |
| --- | --- | --- |
| **Province** | **F** | **M** |
| **Markazi** | 0 | 0.011 |
| **Gilan** | 0 | 0.035 |
| **Mazandaran** | 0.004 | 0.063 |
| **Azerbaijan_East** | 0.002 | 0.015 |
| **Azerbaijan_West** | 0.004 | 0.051 |
| **Kermanshah** | 0 | 0.025 |
| **Khuzestan** | 0 | 0.014 |
| **Fars** | 0.004 | 0.064 |
| **Kerman** | 0.002 | 0.028 |
| **Khorasan_Razavi** | 0.001 | 0.016 |
| **Isfahan** | 0.002 | 0.033 |
| **Sistan&Baluchistan** | 0 | 0.004 |
| **Kurdistan** | 0.008 | 0.062 |
| **Hamadan** | 0.003 | 0.036 |
| **ChaharM&Bakhtiari** | 0 | 0.024 |
| **Lorestan** | 0 | 0.015 |
| **Ilam** | 0 | 0.015 |
| **Kohgiluyeh&BoyerA** | 0.005 | 0.026 |
| **Bushehr** | 0.006 | 0.038 |
| **Zanjan** | 0 | 0.015 |
| **Semnan** | 0.005 | 0.025 |
| **Yazd** | 0 | 0.012 |
| **Hormozgan** | 0 | 0.026 |
| **Tehran** | 0.008 | 0.032 |
| **Ardabil** | 0 | 0.027 |
| **Qom** | - | - |
| **Qazvin** | 0 | 0.095 |
| **Golestan** | 0 | 0.047 |
| **Khorasan_North** | 0 | 0.037 |
| **Khorasan_South** | 0.005 | 0 |
| **Alborz** | 0.004 | 0.039 |
